# Stepwise and Controllable Synthesis of Mesoporous Heterotrimetallic Catalysts Based on Predesigned Al_4_Ln_4_ Metallocycles

**DOI:** 10.1002/advs.202305833

**Published:** 2023-11-16

**Authors:** Dan Luo, Chen‐Hui Liu, Yi‐Bo Chen, San‐Tai Wang, Wei‐Hui Fang, Jian Zhang

**Affiliations:** ^1^ State Key Laboratory of Structural Chemistry Fujian Institute of Research on the Structure of Matter Chinese Academy of Sciences Fuzhou Fujian 350002 P. R. China

**Keywords:** catalysis, compounds, coordination assembly, cyanosilylation, heterometallic materials, porous frameworks

## Abstract

The motivation for making heterometallic compounds stemmed from their emergent synergistic properties and enhanced capabilities for applications. However, the atomically precisely controlled synthesis of heterometallic compounds remains a daunting challenge of the complications that arise when applying several metals and linkers. Herein, a stepwise and controlled method is reported for the accurate addition of second and third metals to homometallic aluminum macrocycles based on the synergistic coordination and hard‐soft acid‐base theory. These heterometallic compounds showed a good Lewis acid catalytic effect, and the addition of hetero‐metals significantly improved the catalytic effect and rate, among that the conversion rate of compound AlOC‐133 reached 99.9% within half an hour. This method combines both the independent controllability of stepwise assembly with the universality of one‐step methods. Based on the large family of clusters, the establishment of this method paves the way for the controllable and customized molecular‐level synthesis of heterometallic materials and creates materials customized for preferential application.

## Graphical abstract

Here, a stepwise controllable method is developed to construct materials customized for the preferential catalytic application. Mesoporous heterotrimetallic framework compounds are obtained using a predesigned Al_4_Ln_4_ heterodimetallic molecular ring. In addition, the catalytic effect on aldehydes is progressively improved by structurally introducing rare‐earth metals and surface ligand modifications, as well as by introducing a third metal to generate a mesoporous structure.

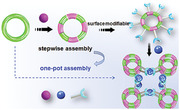

## 1 Introduction

Crystalline heterometallic compounds are compositional diverse and functional complexes allowing performances superior to their parent compound and arising synergistic properties.^[^ ^1^, ^2^ ^]^ In addition, the accurate and periodic distribution of metal ions throughout a lattice of crystalline compounds enables the customized atomically precise structural regulation for specific performance, which could not realized in macroscopic nanomaterials. So far, the developed mixed‐metal approach mainly includes one‐step self‐assembly based on hard‐soft acid‐base theory,^[^ ^3^, ^4^, ^5^, ^6^, ^7^, ^8^ ^]^ and post‐synthetic metalation (redox).^[^ ^1^, ^9^, ^10^, ^11^ ^]^ The first method involves the use of mixed‐donor ligands that discriminate between two types of metal ions through differential binding affinities. The latter post‐synthetic metalation aims to improve performance by adding other metals to the parent homometallic structures. In addition to these two methods, there is an uncommon stepwise method of using metal clusters as raw materials and then combining them with target metals. One successful example is the employ of Cr_3_ oxo cluster to make a series of heterometallic frameworks.^[^ ^12^, ^13^ ^]^ This approach overcomes the limitation of complications arising from the competitive reaction of multiple metals with ligands in a one‐pot synthesis. In practice, however, it requires stable, dissolvable predesigned building blocks making it a daunting challenge and relatively unexplored.

**Scheme 1:**
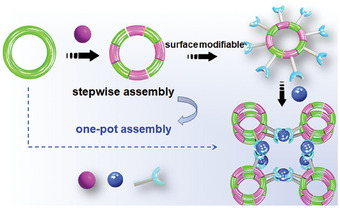
The synthetic strategy toward the mesoporous heterometallic compounds.

Cluster chemistry is an effective way to solve the problem of controllable preparation of heterometallic materials. By studying the predesigned cluster two‐step assembly behavior, we can reverse and generalize to the one‐step synthesis method, which combines the advantages of independent control of the stepwise method and the exploration range and rate of one‐step method (**Scheme** **1**). Based on these considerations and our research on rare earth and aluminum oxo clusters,^[^ ^14^, ^15^, ^16^ ^]^ we report one/two‐step synthesis of heterotrimetallic compounds based on predesigned Al_4_Ln_4_ metallocycles as high‐performance Lewis acid catalysts. Our stepwise design thinking includes: (1) First introducing rare earth metal ions with strong Lewis acid on the aluminum molecular ring to synthesize the heterometallic ring; (2) Modify the surface ligand of the heterometallic ring to manufacture coordination anchors and Lewis base sites; (3) Use coordination‐drive self‐assembly with the pre‐modified heterometallic ring incorporating the third metal ion; (4) Establish a general, controllable one‐step method that can be applied to more modified ligands and a broad range of other third metals. Through the step‐by‐step structural regulation of introducing rare earth metals, modifying surface ligands, and introducing third metals, we have successfully achieved a gradual “structural manufacturing” and stepwise improvement in the catalytic efficiency in cyanosilylation of aldehydes.

## 2 Results and Discussion

### 2.1 Synthesis and Characterization

**Figure 1:**
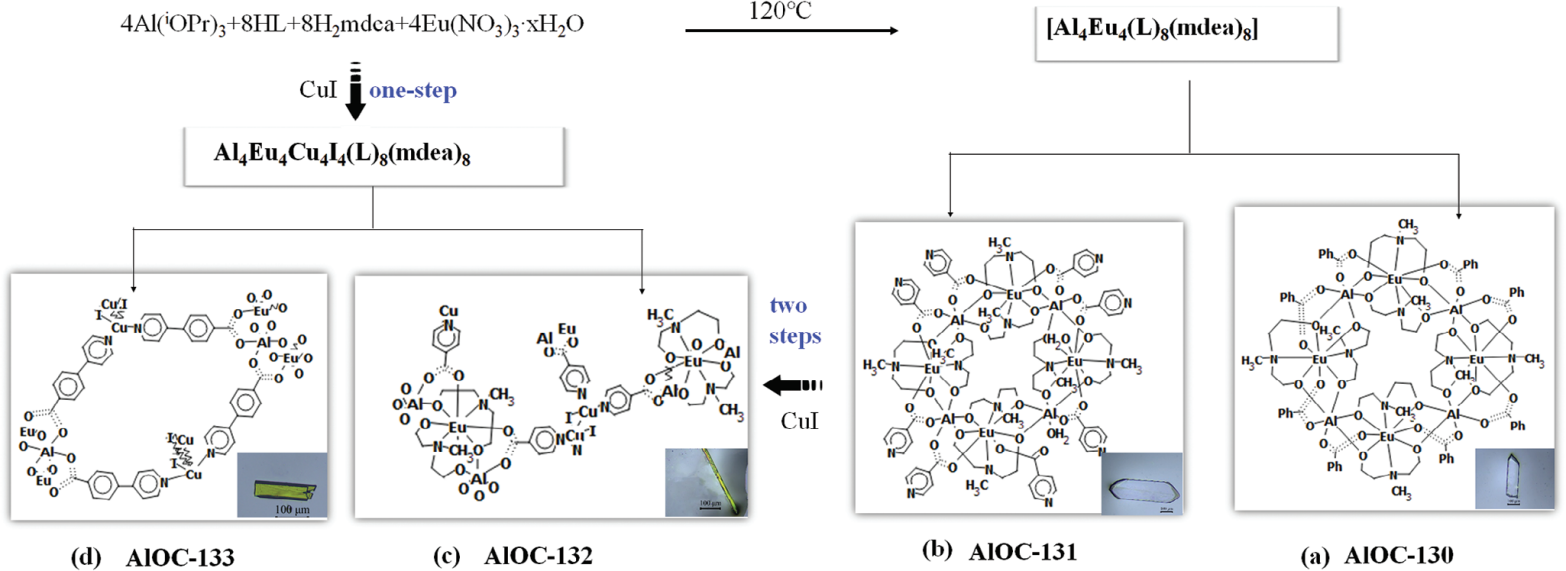
One‐step and two‐step synthesis strategy of heterometallic pore structures based on predesigned ring compounds. (The inset is a photograph of their crystals under a microscope, the scale bar is 100 µm).

A series of heterobimetallic rings and corresponding heterotrimetallic framework compounds were synthesized via amino‐polyalcohol solvothermal synthesis. Rare‐earth‐metal ions are well‐known as hard Lewis acids and N‐methyldiethanolamine (H_2_mdea) has proven to be an effective mixed‐donor ligand widely used in the synthesis of its heterometallic properties.^[^ ^17^, ^18^, ^19^ ^]^ Herein, aluminum isopropoxide, europium nitrate, and sodium benzoate were sonicated in a 1:1:2 stoichiometric ratio in a H_2_mdea/DMF mixture to obtain a colorless clarified solution. The reaction was carried out at 120 °C for 4 days to obtain colorless blocky crystals of compound Al_4_Eu_4_(BA)_8_(mdea)_8_ (**AlOC‐130**) (**Figure** **1**a), and the reaction system always remained clarified (Figure S1a, Supporting Information). We systematically investigated the effects of reaction time and temperature on crystal yield and morphology. It was found that a large number of microcrystals started to appear after 36 h of reaction (Figure S1b, Supporting Information), and the yield of crystals increased rapidly between 72 and 96 h of reaction time (Figure S2, Supporting Information). Its crystallinity was also studied in the temperature range of 80 to 120 °C (Figure S3, Supporting Information). The results show that the reaction starts at 90 °C with the precipitation of a small number of crystals (Figure S4, Supporting Information).

In order to verify the universality of the synthesis of this type of cluster and meanwhile as a precursor for later coordination‐driven assembly, we replaced benzoate with isonicotinic acid (HIN) and successfully isolated colorless strip crystals of compound [Al_4_Eu_4_(IN)_8_(mdea)_8_(H_2_O)]·2H_2_O (**AlOC‐131**) (Figure 1b). In order to prefabricate sufficient clusters as precursors, we performed scale‐up synthesis experiments and managed to obtain 1.12 g of crystal samples for a one‐batch reaction (Figure S5, Supporting Information). Trace amounts of organic amines play an important role in such heterometallic ring reaction systems reported.^[^ ^18^, ^20^, ^21^, ^22^ ^]^ It is worth noting that the synthesis of infinite structures based on heterometallic clusters can be obtained by a two‐step method using prefabricated clusters as precursors and a one‐step method (Figure 1). Copper is selected as the third metal due to its well‐known coordination tendency and affinity toward nitrogen donors. When cuprous was introduced into the mother liquor of **AlOC‐131**, we successfully obtained yellow needle‐like crystals of Al_4_Eu_4_Cu_4_I_4_(IN)_8_(mdea)_8_ (**AlOC‐132**). After verifying that this stepwise coordination‐driven self‐assembly method is feasible, we tried a one‐pot synthesis method to obtain a higher yield of **AlOC‐132**. Another expansion example is the isolation of yellow columnar crystals of Al_4_Eu_4_Cu_4_I_4_(pyba)_8_(mdea)_8_ (**AlOC‐133**, Hpyba = 4‐(4‐pyridyl) benzoic acid). Diversification of synthesis methods toward cluster‐based heterometallic materials paves the way for in‐depth studying of their properties.

**AlOC‐130** and **AlOC‐131** are stable in the air for up to one year, which may be related to the protection of organic ligand shells and the immobilization of alcohol amines with multiple chelation sites (Figures S6 and S7, Supporting Information). In addition, the heterometallic rings are thermally stable up to 350 °C, which is a significant improvement over the previously reported aluminum molecular rings (Figures S15–S20, Supporting Information).^[^ ^15^ ^]^ All the above compounds are stable in organic solvents (Figures S12–S14 and Tables S1,S2, Supporting Information). The generalizability of the compounds toward heavy rare earth ions was confirmed by powder X‐ray diffraction (PXRD) and Fourier transform infrared (FT‐IR) of the isomeric structures (Figures S10,S11 and S21–S24, Supporting Information). As shown in Figures S25–S28 (Supporting Information), the presence of cuprous ions significantly narrows the bandgap of the compound (3.4–1.8 eV change in bandgap from colorless to yellow crystals). The presence of multi‐metal centers in the compounds was confirmed by energy dispersive spectroscopy (EDS) (Figures S29–S32, Supporting Information) and their atomically precise structural information has been unambiguously revealed by single crystal X‐ray diffraction (SCXRD) (**Table** **1**). The EDS‐mapping patterns of the heterometallic compounds show a uniform distribution of Al, Ln, Cu, C, N, O, and I atoms in the crystals (Figures S33–S36, Supporting Information). The bond valence sum (BVS) indicates that the valence states of the heterotrimetallic Al, Eu, and Cu in the compound are 3+, 3+, and 1+, respectively (Tables S3–S6, Supporting Information).

**Table 1:** Summary of heterometallic rings compounds: crystal data and structure refinement results.

compounds	formula^a)^	sp.gr.	*a* (Å)	*b* (Å)	*c* (Å)	*V* (Å)	*R*_int_ ^b)^	CCDC^c)^
**AlOC‐130**	Al_4_Eu_4_(BA)_8_(mdea)_8_	*P*‐42_1_c	23.3687(10)	23.3687(10)	9.8818(10)	5396.41(7)	0.0386	2288338
**AlOC‐131**	[Al_4_Eu_4_(IN)_8_(mdea)_8_ (H_2_O)]·2H_2_O	*P*2_1_/n	18.9563(2)	21.8313(2)	27.7034(4)	11447.5(2)	0.0592	2288339
**AlOC‐132**	Al_4_Eu_4_Cu_4_I_4_(IN)_8_(mdea)_8_	*I*4/mmm	31.674 (4)	31.674 (4)	25.087 (5)	25169(7)	0.0998	2288340
**AlOC‐133**	Al_4_Eu_4_Cu_4_I_4_(pyba)_8_(mdea)_8_	*C*mcm	41.3594(17)	17.9180(8)	40.8506(15)	30273(2)	0.1618	2288341

^a)^ Abbreviations: BA = benzoic acid; IN = isonicotinic acid; mdea = N‐methyldiethanolamine; pyba = 4‐(4‐pyridy)benzoic acid;

^b)^ Crystallographic data of the structures were solved with direct methods using OLEX2 v1.2 © OlexSys Ltd. 2004 – 2023. Detailed X‐ray crystallographic data are provided in Tables S10,S11 (Supporting Information);

^c)^ CCDC numbers are applied from the Cambridge Crystallographic Data Centre database. The crystal under investigation showed no significant intensity at a high angle and the increasing flexibility of the lengthened carbon chain compromising the data quality and the model obtained.

2.2

**Figure 2:**
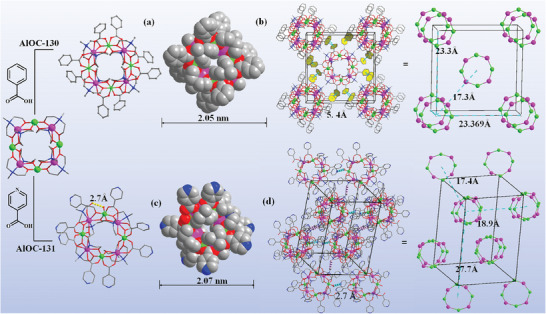
Molecular structures of heterobimetallic molecular rings. a) Ball‐and‐stick and space‐filling modeling diagram of the compound **AlOC‐130**. b) Stacking diagram of the compound **AlOC‐130** c) Ball‐and‐stick and space‐filling modeling diagram of the compound **AlOC‐131**. d) Stacking diagram of the compound **AlOC‐131**. Hydrogen atoms are omitted for clarity. Color code: Al, green; Eu: pink; C, gray; O, red; N, and blue. Black lines represent unit cells. Non‐metal atoms have been omitted for clarity of the schematic.

Compound Al_4_Eu_4_ is heterobimetallic molecular ring crystallizing in the tetragonal space group *P*‐42_1_c (**Figure** **2**a). The neutral octanuclear ring consists of an alternating arrangement of four Al^3+^ and four Eu^3+^ ions bridged by eight fully deprotonated mdea^2−^ and eight benzoates (Figure S38a, Supporting Information). Such alternating arrangement is different from the “Dy_4_‐square‐within‐a‐Ga_4_‐square”,^[^ ^23^ ^]^ “Tower‐Like” Ln_4_Cr_4_^[^ ^24^ ^]^ and the square [Fe_4_Gd_4_],^[^ ^23^ ^]^ but is similar to the wheel‐like Sc_4_Gd_4_ (Figures S39–S41, Supporting Information).^[^ ^18^ ^]^ Compared with the boat‐shape side‐view of the Sc_4_Gd_4_, Al_4_Eu_4_ can be viewed as a chair‐shape with a dihedral angle of 40.17^o^ (Figure S38b, Supporting Information). Space‐filling diagram of Al_4_Eu_4_ reveals that the size of this molecule is ca. 2.1 nm in length and ca. 0.8 nm in thickness (Figure 2a).

The two pincer‐like mdea^2−^ ligands chelate one rare earth ion and benzoic acid further connects Al and Eu ions. Each Al^3+^ takes the standard six‐connected octahedral geometry (Figure S42a, Supporting Information), consisting of oxygen from two carboxylic acid ligands and four mdea^2−^ (Figure S42b, Supporting Information). Each rare earth ion, on the other hand, is octa‐ligated consisting of two N and six O (Figure S42c, Supporting Information), and they come from two mdea^2−^ that take the µ_3_‐η^2^:η^1^:η^2^ coordination pattern and two carboxylic acid ligands that take the µ_2_‐η^1^:η^1^ coordination pattern (Figure S42d,e, Supporting Information). As shown in Figure 2b, the heterobimetallic molecular rings are stacked in tetragonal arrays through *π*–*π* interactions of aromatic ligands (Figure S44, Supporting Information). The distance between the heterometallic rings in the unit cell ranges from 9.88–23.67 Å (Figure S45a, Supporting Information). The total solvent‐accessible volumes of **AlOC‐130** as calculated by PLATON are 5.1%.

Bifunctional isonicotinic acid linkers were introduced as both potential coordination anchors for the subsequent coordination assembly (pyridine nitrogen (*N*_py_) coordination sites) and potential adsorption sites for catalytic substrates. The isonicotinic acid‐modified compound **AlOC‐131** (Figure 2c) crystallizes in the monoclinic space group *P2_1_/n*. The reduced symmetry is due to a local change in the coordination environment of the Al ions. As shown in Figure 2d, there is terminal isonicotinic acid and the nearest vacancy is occupied by a water molecule, generating strong hydrogen bonding interactions (O—H—O, 2.657 Å) within the molecular ring (Figure S47, Supporting Information). Instead of the *π*–*π* interactions, these rings are interconnected by hydrogen bonding interactions ranging from 2.71 to 3.40 Å (Figure S47d; Table S8, Supporting Information). The distances between the heterometallic rings in the unit cell of the compound **AlOC‐131** were in the interval 17.36–27.60 Å (Figure S45b, Supporting Information) and the total solvent‐accessible volumes of **AlOC‐131** as calculated by PLATON are 14.1%.

2.3

**Figure 3:**
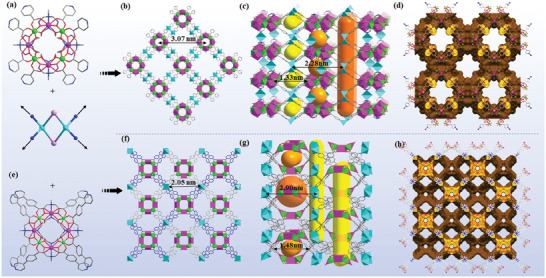
The molecular structures of mesoporous heterotrimetallic compounds. Ball‐and‐stick diagram of the {Al_4_Ln_4_} unit in the a) compound **AlOC‐132,** and e) compound **AlOC‐133**. Stacking diagram of b) compound **AlOC‐132** and f) compound **AlOC‐133** along [001] direction. Different colors indicate different layers of the interspersed structure. Cavities in the c) compound **AlOC‐132** and g) compound **AlOC‐133**. Perspective view of void space of d) compound **AlOC‐132** and h) compound **AlOC‐133** along *c*‐axis. Hydrogen and some non‐metal atoms are omitted for clarity. Color code: Al, green; Eu: pink; Cu, blue; I: violet; C, gray; O, red; N, blue.

Compound **AlOC‐132** is a mesoporous 3D framework consisting of above mentioned similar Al_4_Eu_4_ heterometallic ring with Cu_2_I_2_ units (**Figure** **3**; Figures S48,S49, Supplementary Movie S1). Notably, aluminum's defect site in the pristine discrete cluster of **AlOC‐131** disappeared and its connections are obviously different from our previously reported homometallic Al_8_ ring reducing from 12 to 8 (Figure S50, Supporting Information).^[^ ^25^ ^]^ Through the use of coordination‐driven self‐assembly, we isolated the infinite porous compound **AlOC‐132** derived from **AlOC‐131** cluster precursor. The eight isonicotinic acids on each heterobimetallic ring are connected to the surrounding eight heterobimetallic rings via Cu_2_I_2_ units to generate a 4,8‐connected *scu* net (Figure S51, Supporting Information). Mesoporous 1D channels, relatively small channels and microporous cages co‐exist in the structure (dimensions 3.07^*^3.07 nm^2^, 1.25^*^2.28^*^2.28 nm^3^ and 1.33^*^1.09 nm^2^, respectively) (Figure 3c,d). The type I square channel comprises heterometallic rings at the four vertices and four Cu_2_I_2_ on the prongs connected by ligands running along the *c*‐axis (Figure S53a, Supporting Information). The type II rhombic channel interleaved with the type I channel is made up of alternating heterometallic rings and Cu_2_I_2_ sections distributed at the apex running along the *b*‐axis (Figure S53b, Supporting Information). The type III basket‐liked cavity involves two heterometallic rings at the apex and four Cu_2_I_2_ sections at the waist (Figure S53c, Supporting Information). The distance between the heterometallic rings in the porous framework varied from 12.54 to 22.40 Å (Figure S54, Supporting Information). The total solvent‐accessible volumes of **AlOC‐132** as calculated by PLATON are 61.8%.

To verify the universality of the assembly method and obtain an expanded pore structure, we introduced an elongated version of the Hpyba ligand. However, the result turned out that compound **AlOC‐133** was isolated in the form of a stable double‐interpenetrated version due to the lack of suitable support for such macropores (Figure 3). Nevertheless, the coordination‐driven self‐assembly did work well. The use of lengthened flexible pyba^−^ maintains the same number of connections as that in **AlOC‐132**, albeit with an increase in the dihedral angle of the inorganic {Al_4_Ln_4_} cluster (from 29.41^o^ to 47.76^o^) (Figures S55,S56, Supporting Information). The elongated version of the organic ligand with flexibility on each heterobimetallic ring undergoes torsion to connect to the other eight heterobimetallic rings to form an interpenetrating 4,8‐connected 2 (1 + 1) interpenetrating *scu* net (Figures S58–S60, Supplementary Movie S2). It should be noted that the situation of cavities changes accordingly (Figure 3g). First, the large square channel with dimensions of 4.05^*^4.05 nm^2^ was divided into four smaller channels with sizes of 2.05^*^2.05 nm^2^ (Figure S61a, Supporting Information). Second, the size of the microporous channels is smaller (size 0.92^*^1.48 nm^2^ vs 1.33^*^1.09 nm^2^) (Figure S61b, Supporting Information). Then, the cage cavity is wider in dimension (size 1.25^*^2.28^*^2.28 nm^3^ vs 1.82^*^2.90^*^2.90 nm^3^) (Figure S61c, Supporting Information). PLATON calculations reveal that the porosity of **AlOC‐133** (64.1%) is still slightly higher than that of **AlOC‐132** (61.8%) even though it is interpenetrated. Hence, we establish a controllable synthesis route toward a specific topological framework. These channels and pores in compounds **AlOC‐132** and **AlOC‐133** are sufficient to accommodate the benzaldehyde substrate (4.34^*^2.43 Å^2^) in the subsequent catalytic process.

### 2.4 The Catalytic Activity of Heterometallic Ring Compounds

The cyanylation of carbonyl compounds with TMSCN, a typical Lewis acid‐catalyzed reaction, is an important reaction in organic synthesis for the formation of C—C bonds to produce cyanohydrin derivatives.^[^ ^26^, ^27^, ^28^, ^29^ ^]^ While there are many materials used to catalyze this reaction including organic small molecules,^[^ ^30^, ^31^ ^]^ metal complexes^[^ ^32^, ^33^, ^34^ ^]^, and crystalline materials,^[^ ^35^, ^36^ ^]^ among them crystalline materials with well‐defined structural information can provide insight into the catalytic mechanism at the atomic level. Crystalline materials are mainly focused on metal‐organic frameworks (MOFs),^[^ ^37^ ^]^ covalent organic frameworks (COFs)^[^ ^38^ ^]^, and polyoxometalates (POMs).^[^ ^39^ ^]^ However, much of the work reported so far shows the structure of the catalyst or a single metal as the active site, neither of which provides insight into the catalytic mechanism. In our previous work, the binding of substrates to the aluminum‐based molecular rings during catalysis was tried and successfully confirmed.^[^ ^40^ ^]^ Considering the abundance of metal nodes (Al, Eu, and Cu as Lewis acid sites) and the porous nature of these heterometallic rings and their framework materials, they are potential Lewis acid catalysts and shed more light on the reaction from a multi‐metallic synergistic perspective. Hence, we chose cyanylation of benzaldehyde as a typical probe for Lewis acid‐catalyzed reactions.

**Figure 4:**
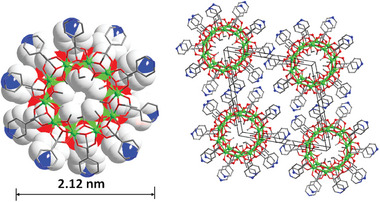
The molecular structure and supramolecular packing diagram of AlOC‐79.

Quantitative product yields were obtained by catalyzing the reaction of benzaldehyde (0.5 mmol) with TMSCN (1 mmol) in CH_2_Cl_2_ with 1.5 mol.% of AlOCs catalyst loading at room temperature under inert conditions for 2 h (**Table** **2**). As shown in Table 2 entries 1–3, aromatic ligands alone have little effect on the reaction (Figure S62, Supporting Information). To clarify the catalytic active center, we synthesized a structural similar homometallic molecular ring **AlOC‐79** modified by isonicotinic acids (**Figure** **4**; Figure S63, Supporting Information). Compound **AlOC‐79** is a ten‐membered ring with an organic shell environment similar to **AlOC‐131**. We can see from the catalytic result that it did not catalyze as well as either of the heterometallic rings (**AlOC‐130** to **AlOC‐133**), suggesting that the introduction of lanthanide metal ions as Lewis acid sites enhances the catalysis (Table 2 entry 4). The catalytic effects of the heterometallic molecular rings and their framework were in the order of **AlOC‐130**< **AlOC‐131**< **AlOC‐132**< **AlOC‐133** (88.0%, 95.5%, 98.2%, and 99.9%, respectively) (Table 2 entries 5–8). It is worth mentioning that the reaction was highly selective and no by‐products were observed (Figures S64–S67, Supporting Information). The improved effect of **AlOC‐131** compared with **AlOC‐130** resulted from the presence of N‐substituted aromatic ring that facilitates binding the substrate to the catalyst. In addition, the coordination mode of the organic ligand also affects the catalytic reaction to some extent.^[^ ^35^, ^41^ ^]^ As described in the structure section and shown in Figure S68 (Supporting Information), the emergence of a terminal isonicotinic and the local defect of the attack of water molecules on Al ions make it increase the Lewis base site and easier to contact with the substrate. The isostructural lanthanide series **AlOC‐131‐Ln** has a considerable catalytic effect, indicating the synergistic effect of Lewis acid site and surface ligand modification (Figure S69, Supporting Information).

**Table 2:** Comparison of benzaldehyde cyanosilylation reactions catalyzed by different catalysts.

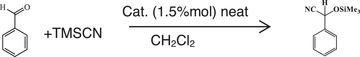			
Entry^a)^	Catalytic	Time (h)	Yield (%)^b)^
1	HIN^c)^	2	None
2	Hpyba^d)^	2	None
3	BA^e)^	2	None
4	AlOC‐79^f)^	2	70.1
5	**AlOC‐130**	2	88.0
6	**AlOC‐131**	2	95.5
7	**AlOC‐132**	2	98.2
8	**AlOC‐133**	0.5	99.9

^a)^ Reaction conditions: catalyst 1.5 mol%, aldehyde 0.5 mmol, TMSCN 1 mmol, and CH_2_Cl_2_ as solvent, temperature (303 K) under N_2_;

^b)^ Yields were determined by ^1^H NMR using CH_2_Br_2_ as an internal standard;

^c)^ HIN = isonicotinic acid;

^d)^ Hpyba = 4‐(4‐pyridy) benzoic acid;

^e)^ BA = benzoic acid;

^f)^ A ring of aluminum molecules protected by isonicotinic acid and ethoxy (Figure 4, Figure S63, Supporting Information).^[^ ^25^ ^]^

The substrate range for the aldehyde cyanosilylation reaction was investigated using **AlOC‐130** as an example (Figure S70, Supporting Information). Under standard conditions, when aromatic aldehydes with electronic effect substituents (electron‐donating ‐OCH_3_, electron‐withdrawing ‐CF_3_) or heterocyclic aldehydes were employed, the corresponding products were obtained in high yields after 2 h (85%–95%), which suggests that the reaction is broad tolerance to various substrates. However, 1‐naphthaldehyde and ketone obtained lower catalytic efficiency even after prolonged reaction time (24 h) (30%–40%), which may be related to the spatial site resistance of the substrates.

Moreover, the catalytic effect of the heterotrimetallic compounds **AlOC‐132** and **AlOC‐133** are superior to those of the heterometallic molecular rings **AlOC‐130** and **AlOC‐131** not only in terms of yield but also in the rate of conversion, which is better illustrated by the presence of the pores structures and triple‐metal centers (Figure S71, Supporting Information).^[^ ^36^, ^38^, ^42^ ^]^ One possible reason for the rapid complete conversion (0.5 h) of the substrate catalyzed by the compound **AlOC‐133** includes the highest porosity throughout these series of compounds (5.1%, 14.1%, 61.8%, and 64.1% for **AlOC‐30** to **AlOC‐133**, respectively).^[^ ^37^ ^]^ Another reason is the presence of more regular 1D channels and favorable cavity environments (abundant aromatic walls and iodine atoms pointing toward the channel favor the formation of *π*–*π* interactions and O—H—I hydrogen bonding, respectively, with the substrate benzaldehyde) (Figures S72–S75, Supporting Information). To investigate the Lewis acid properties of the heterometallic ring and its framework compounds, we performed pyridine FT‐IR spectroscopy. As shown in Figure S76 (Supporting Information), the pyridine FT‐IR pattern exhibited three signal peaks at 1039, 1047, and ≈1059 cm^−1^ corresponding to the adsorption of pyridine on the Lewis acid sites.^[^ ^43^, ^44^, ^45^, ^46^ ^]^ As expected, the amount of Lewis acid sites increases with the order of catalytic effect. Hence, the synergy of the multiple Lewis acid sites, the pore cavity and the microenvironment are conducive to a good catalytic effect. Their performances are significantly better than those of lanthanide‐based polyoxometalates and MOFs (Table S9, Supporting Information),^[^ ^39^, ^47^, ^48^ ^]^ although their catalytic effect is inferior to those of monomer organo‐aluminum with flexible active sites.^[^ ^30^, ^49^, ^50^ ^]^

## 3 Conclusion

In summary, we have demonstrated the stepwise and controllable synthesis of mesoporous heterotrimetallic compounds based on predesigned metallocycles. This synthesis strategy can be extended to a broad range of metals and a handful of bifunctional linkers like pyrazolecarboxylate, imidazolecarboxylate, etc and their derivatives. It is worth mentioning that multi‐metallic centers in heterometallic rings and their framework compounds can serve as Lewis acid sites and are potentially excellent catalysts. Among them, the heterometallic molecular ring exhibits better catalytic activity compared to the homometallic aluminum molecular ring. And the porous heterotrimetallic framework with regular 1D channels, abundant aromatic walls, and larger cavity sizes showed a superior catalytic effect than the heterobimetallic molecular ring. This work provides methods to guide heterometallic molecular ring synthesis, surface modification, and designable assembly to produce porous framework materials and contributes to understanding catalytic reaction mechanisms from various perspectives of multi‐metallic centers, pore environments. In addition, such porous polymetallic frameworks may have broad applications in selective separation and photo(electro)catalysis.

## 4 Experimental Section

### Materials and Methods

All the reagents and solvents were purchased commercially and were used without further purification. Aluminum isopropoxide (Al(O^i^Pr)_3_) and methylamine ethanol solution (40%, 120 µL) were acquired from Aladdin Chemical Reagent Shanghai. N‐propyl alcohol (HO^n^Pr), N, N‐dimethylformamide (DMF), and sodium benzoate was bought from Sinopharm Chemical Reagent Beijing. Isonicotinic acid (HIN), N‐methyldiethanolamine (H_2_mdea) were purchased from Adamas‐beta. 4‐(4‐pyridy) benzoic acid (Hpyba) was acquired from Jilin Chinese Academy of Sciences‐Yanshen Technology Co., Ltd.

### Synthesis of the Predesigned Heterobimetallic Clusters and Extended Porous Heterotrimetallic Networks

Colorless strips **AlOC‐130** crystals were synthesized by amino‐polyalcohol solvothermal synthesis reaction of aluminum isopropoxide (204 mg, 1 mmol), europium nitrate hexahydrate (60 mg, 0.13 mmol), and sodium benzoate (150 mg, 1 mmol) in a solvent mixture containing N‐methyldiethanolamine (2.5 mL) and DMF (2.5 mL) at 120 °C for 4 days. When sodium benzoate was substituted with isonicotinic acid, colorless bulk crystals of compound **AlOC‐131** were isolated. Yellow needle‐like **AlOC‐132** and yellow columnar crystals of **AlOC‐133** were obtained by adding n‐propanol suspension of cuprous iodide to the above system by HIN and lengthened Hpyba ligand, respectively. For a more detailed synthesis process please refer to the Electronic Supporting Information (ESI).

### X‐ray Crystallography

Single crystal X‐ray diffraction data of AlOCs were collected on Hybrid Pixel Array detector equipped with Ga‐Kα radiation (λ = 1.3405 Å) at about 100 K. The structures were solved with the dual‐direct methods using ShelxT and refined with the full‐matrix least‐squares technique based on F^2^ using the SHELXL.^[^ ^51^ ^]^ Non‐hydrogen atoms were refined anisotropically. Hydrogen atoms were added theoretically, riding on the concerned atoms and refined with fixed thermal factors. All absorption corrections were performed using the multi‐scan program. The crystals of the compounds **AlOC‐132** and **AlOC‐133** are so small that high angle diffraction is weak. Some of the atoms on the chelating ligand N‐methyldiethanolamine could not be fixed. Despite many attempts, it ended up with failure. Their presence has also been confirmed by a variety of other characterizations including FT‐IR, EDS, and so on. The obtained crystallographic data are summarized in Tables S10,S11 (Supporting Information).

### General Procedure of Cyanosilylation

The mixture of aldehyde, trimethylsilyl cyanide (TMSCN) and CH_2_Cl_2_ was added to the schlenk tube (0.5 mmol aldehyde, 1 mmol TMSCN and 5 mL CH_2_Cl_2_), where AlOCs had been introduced in advance. The mixture was stirred (200 rpm) at room temperature for 2 h, under N_2_ atmosphere. Yields were determined by ^1^H‐NMR analysis using CH_2_Br_2_ as an internal standard.

## Conflict of Interest

The authors declare no conflict of interest.

## Supporting information

Supporting Information

Supplementary Movie 1

Supplementary Movie 2

## Data Availability

The data that support the findings of this study are available from the corresponding author upon reasonable request.
