# Supplementary material for: Stepwise and Controllable Synthesis of Mesoporous Heterotrimetallic Catalysts Based on Predesigned Al4Ln4 Metallocycles
Source: Adv Sci (Weinh). 2023 Nov 16;11(2):2305833. doi: 10.1002/advs.202305833 (PMC10787057; doi:10.1002/advs.202305833)
Supplement: Supplementary file 1 — Supporting Information [file ADVS-11-2305833-s001.pdf]

ADVANCED SCIENCE Open Access

Supporting Information

for *Adv. Sci.*, DOI 10.1002/advs.202305833

Stepwise and Controllable Synthesis of Mesoporous Heterotrimetallic Catalysts Based on Predesigned Al_4Ln_4 Metallocycles

Dan Luo, Chen-Hui Liu, Yi-Bo Chen, San-Tai Wang, Wei-Hui Fang and Jian Zhang*

Supplementary Information

Stepwise and Controllable Synthesis of Mesoporous Heterotrimetallic Catalysts Based On Predesigned Al_4Ln_4 Metallocycles

Dan Luo, Chen-Hui Liu, Yi-Bo Chen, San-Tai Wang, Wei-Hui Fang* and Jian Zhang

State Key Laboratory of Structural Chemistry

Fujian Institute of Research on the Structure of Matter,

Chinese Academy of Sciences, Fuzhou, Fujian 350002, P. R. China.

E-mail: fwh@fjirsm.ac.cn

Content

1. Supplementary Notes. S3

1.1 Chemicals and Materials. S3
1.2 Energy dispersive spectroscopies (EDS). S3
1.3 Fourier Transform Infrared (FT-IR) Spectroscopies S3
1.4 UV-vis spectroscopies. S3
1.5 Powder X-ray diffractions (PXRD). S3
1.6 Thermogravimetric analyses. S3
1.7 Nuclear magnetic spectrum. S3
1.8 General procedure of AlOCs-catalyzed cyanosilylation S4
1.9 Crystal photos. S4
1.10 Drawing softwares S4
1.11 General methods for X-ray Crystallography S4

2. Supplementary Methods. S5

2.1 Synthesis of heterometallic rings. S5
2.2 Assembly of Al-Ln heterometallic molecular rings. S5
2.3 Assembly of Al-Ln heterometallic molecular rings using elongated ligands S6

3. Supplementary Figures. S7

3.1 Syntheses. S7
3.2 PXRD analysis for heterometallic compounds. S8
3.3 Stability of heterometallic compounds. S10
3.4 TGA test for heterometallic comheterometallic compounds. S12
3.5 FT-IR spectra for heterometallic compounds. S14
3.6 The solid-state absorption spectra of heterometallic compounds. S16
3.7 EDS spectra for heterometallic compounds. S17
3.8 EDS-mapping spectra for heterometallic compounds. S19
3.9 Detailed structure information for heterometallic rings. S20

3.10 Heterogeneous catalytic activity of heterometallic compounds. S29
4. Supplementary Tables.. S34
4.1 The solubility of heterometal rings in different solvents S34
4.2 Bond valence sum (BVS) calculations of heterometal rings. S35
4.3 Hydrogen bond parameters.. S37
4.4 Summary of catalysts used in the catalytic reaction of cyanogenation of aldehydes. S38
4.5 Crystallographic data of heterometal rings. S40
5. Supplementary Reference.... S42

1. Supplementary Notes.

1.1 Chemicals and Materials.

All the reagents and solvents employed are purchased commercially and used as received without further purification. Aluminum isopropoxide ($Al(O^iPr)_3$) and methylamine ethanol solution (40 %, 120 μL) was acquired from Aladdin Chemical Reagent Shanghai. N-propyl alcohol (HO^nPr), N, N-dimethylformamide (DMF), sodium benzoate was bought from Sinopharm Chemical Reagent Beijing. Isonicotinic acid (HIN), N-methyldiethanolamine (H_2mdea) were purchased from Adamas-beta. 4-(4-pyridy) benzoic acid (Hpyba) was acquired from Jilin Chinese Academy of Sciences-Yanshen Technology Co., Ltd.

1.2 Energy dispersive spectroscopies (EDS).

The EDS analyses of single crystals were performed on a JEOL JSM6700F field-emission scanning electron microscope equipped with an Oxford INCA system.

1.3 Fourier Transform Infrared (FT-IR) Spectroscopies.

FT-IR spectra (KBr pellets) were recorded on an ABB Bomem MB102 spectrometer.

1.4 UV-vis spectroscopies.

The UV-vis diffuse reflection data were recorded at room temperature using powder samples with $BaSO_4$ as a standard (100% reflectance) on a PerkinElmer Lamda-950 UV spectrophotometer and scanned at 200-800 nm. The absorption data are calculated from the Kubelka-Munk function, ($F(R) = (1-R)^2/2R$), where R represents the reflectance.

1.5 Powder X-ray diffractions (PXRD).

Single crystal samples of compounds **AlOC-130** to **AlOC-133** were grinded in a mortar and sieved with a 200 molybdenum sieve and then used for testing. PXRD data were collected on a Rigaku Mini Flex II diffractometer using Cu-Kα radiation (λ = 1.54056 Å) under ambient conditions. Molecular modeling was carried out using Reflex Plus, a module implemented in Materials Studio (version 4.4) by Accelrys Inc. The initial structures were constructed piecewise starting with a tetragonal cell with space group $P\text{-}42_1c$ for **AlOC-130,** a monoclinic cell with space group $P2_1/n$ for **AlOC-131**, a tetragonal cell with space group *I*4/mmm for **AlOC-132** and orthorhombic cell with space group *C*mcm for **AlOC-133**. For **AlOC-130**, the Pseudo-Voigt function was used for whole profile fitting and Berrar-Baldinozzi function was used for asymmetry correction during the refinement processes. For **AlOC-131**, **AlOC-132** and **AlOC-133**, the Tomandl Pseudo-Voigt function was used for whole profile fitting and Berrar-Baldinozzi function was used for asymmetry correction during the refinement processes.

1.6 Thermogravimetric analyses.

The thermogravimetric analyses (TGA) were performed on a Mettler Toledo TGA/SDTA 851e analyzer in a nitrogen atmosphere with a heating rate of 10 ^{o}C/min.

1.7 Nuclear magnetic spectrum.

^{1}H NMR spectra were recorded on a Bruker AVANCE III spectrometer (400 MHz) or a JEOL ECZ600S spectrometer (600 MHz).

1.8 General procedure of AlOCs-catalyzed cyanosilylation.

The mixture of aldehyde, trimethylsilyl cyanide (TMSCN) and CH_2Cl_2 was added to the schlenk tube (0.5mmol aldehyde, 1mmol of TMSCN and 5mL CH_2Cl_2), where the activated AlOCs had been introduced in advance. The mixture was stirred (200 rpm) at room temperature for 2 h, under N_2 atmosphere. Yields were determined by ^{1}H-NMR analysis using CH_2Br_2 as an internal standard.

1.9 Crystal photos.

Crystal photos were obtained on a LW500LJT infinite high-definition transmission-reflection metallographic microscope with a 20-megapixel color imaging system.

1.10 Drawing softwares

The structural diagrams are plotted using Diamond 4.6.5. The channel simulations were realized by using Mercury 2022.3.0. The topology of the multi-metal framework compounds was analyzed by Topos-pro software.

1.11 General Methods for X-ray Crystallography

Crystallographic data of **AlOC-133** was collected on a Supernova single crystal diffractometer equipped with graphite-monochromatic Cu Kα radiation (λ = 1.5418 Å). Crystallographic data of other compounds (**AlOC-130** to **AlOC-132**) were collected on a Rigaku Synergy Custom (Liquid MetalJet D2+) diffractometer with Ga K*α* radiation (λ = 1.3405 Å). The structures were solved with direct methods using OLEX^{2} and refined by full-matrix least-squares on F^2 using *SHELXTL*.[1] All hydrogen atoms were theoretical hydrogenation, riding on the relevant atoms and refined with fixed thermal factors. Non-hydrogen atoms were refined anisotropically. And N-methyldiethanolamine molecules are severely disordered and the related hydrogen atoms were not included in compound **AlOC-132** and compound **AlOC-133**. The crystal of compound **AlOC-132** is extremely tiny, causing it to diffract weakly at high angles. The overall completeness of its crystallographic data is relatively low, but the combination of some other features sufficiently confirms its composition. The obtained crystallographic data for **AlOC-130** to **AlOC-133** are summarized in Supplementary Table 10 to 11.

2. Supplementary Methods.

2.1 Synthesis of heterometallic rings.

Synthesis of $Al_4Eu_4(BA)_8(mdea)_8$ (**AlOC-130**).

A mixture of aluminum isopropoxide (204 mg, 1 mmol), sodium benzoate (150 mg, 1.04 mmol), europium nitrate hexahydrate (60 mg, 0.13 mmol), N-methyldiethanolamine (2.5 mL), DMF (2.5 mL) and methylamine ethanol solution (40 %, 120 μL) was sealed in a 20 mL vial and transferred to a preheated oven at 120 °C for

4 days. No crystals were obtained when sodium benzoate was replaced with benzoic acid, indicating that the sodium ion plays an important role in the reaction system. When cooled to room temperature, colorless blocky crystals were obtained. Among them, europium nitrate hexahydrate can be replaced by samarium nitrate hexahydrate, gadolinium nitrate hexahydrate, terbium nitrate hexahydrate, dysprosium nitrate hexahydrate, holmium nitrate pentahydrate, erbium nitrate pentahydrate, thulium nitrate pentahydrate, yttrium nitrate hexahydrate and lutetium nitrate hexahydrate to obtain colorless, pink and pale yellow crystals, respectively. The crystals are rinsed with DMF and preserved under a sealed and dry environment. (yield: 38.21% based on $Al(O^iPr)_3$). FT-IR (KBr, cm^{-1}): 3050(w), 2963(w), 2880(w), 2840(m), 2362(m), 2325(w), 1592(m), 1552(s), 1408(w), 1307(w), 1091(w). Elemental analysis calcd. (%) for $Al_4Eu_4C_{96}N_8O_{32}H_{104}$ (MW 2597.63): C 44.39, N 4.31, H 4.00; found C 43.31, N 4.54, H 4.86.

Synthesis of $[Al_4Eu_4(IN)_8(mdea)_8(H_2O)]·2H_2O$ (AlOC-131).

A mixture of aluminum isopropoxide (204 mg, 1 mmol), isonicotinic acid (150 mg, 1.25 mmol), europium nitrate hexahydrate (60 mg, 0.13 mmol), N-methyldiethanolamine (2.5 mL), DMF (2.5 mL) and methylamine ethanol solution (40 %, 120 μL) was sealed in a 20 mL vial and transferred to a preheated oven at 120 °C for 4 days. When cooled to room temperature, colorless striped crystals were obtained. Among them, europium nitrate hexahydrate can be replaced by samarium nitrate hexahydrate, gadolinium nitrate hexahydrate, terbium nitrate hexahydrate, dysprosium nitrate hexahydrate, holmium nitrate pentahydrate, erbium nitrate pentahydrate, thulium nitrate pentahydrate, yttrium nitrate hexahydrate and lutetium nitrate hexahydrate to obtain colorless, pink and pale yellow crystals, respectively. The crystals are rinsed with DMF and preserved under a sealed and dry environment. (yield: 40.74% based on $Al(O^iPr)_3$). FT-IR (KBr, cm^{-1}): 3058(s) 2963(w), 2875(w), 2360(m), 2330(m), 1667(w), 1591(m), 1540(s), 1485(w), 1445(w), 1296(w). Elemental analysis calcd. (%) for $Al_4Eu_4C_{88}N_{16}O_{35}H_{120}$ (MW 2677.05): C 39.48, N 8.37, H 4.48; found C 38.05, N 7.90, H 4.56.

Scale-up synthesis of AlOC-131.

A mixture of aluminum isopropoxide (2g, 10 mmol), isonicotinic acid (1.5 g, 12.5 mmol), europium nitrate hexahydrate (600 mg, 1.3 mmol), N-methyldiethanolamine (25 mL), DMF (25 mL) and methylamine ethanol solution (40 %, 1.2 mL) was sealed in a 100 mL vial and transferred to a preheated oven at 120 °C for 4 days. When cooled to room temperature, colorless striped crystals were washed with DMF over and over. After drying, the precipitate and crystals are sieved by 200-mesh sieve, and pure phase striped crystals (~50μm) can be obtained (about 1.12g).

2.2 Assembly of Al-Ln heterometallic molecular rings.

One-step synthesis of $Al_4Eu_4Cu_4I_4(IN)_8(mdea)_8$ (AlOC-132).

A mixture of aluminum isopropoxide (102 mg, 0.5 mmol), isonicotinic acid (150 mg, 1.25 mmol), europium nitrate hexahydrate (60 mg, 0.13 mmol), cuprous iodide (60 mg, 0.32 mmol), N-methyldiethanolamine (2.5 mL), DMF (2.5 mL), n-propanol (2.5 mL) and methylamine ethanol solution (40 %, 120 μL) was sealed in a 20 mL vial and transferred to a preheated oven at 120 °C for 4 days. When cooled to room temperature, yellow needle crystals were obtained. Among them, europium nitrate hexahydrate can be replaced by samarium nitrate hexahydrate, gadolinium nitrate hexahydrate, terbium nitrate hexahydrate, dysprosium nitrate hexahydrate, holmium nitrate pentahydrate, erbium nitrate pentahydrate, thulium nitrate pentahydrate, yttrium nitrate hexahydrate and lutetium nitrate hexahydrate to obtain colorless, pink and pale yellow crystals, respectively. The crystals are rinsed with DMF and preserved under a sealed and dry environment.

(yield: 24.3% based on Al(O^{i}Pr)$_3$). FT-IR (KBr, cm^{-1}): 3055(w), 2963(w), 2886(w), 2843(w), 1594(s), 1537(s), 1488(w), 1451(w), 1394(s), 1258(w), 1091(m).

Stepwise synthesis of $Al_4Eu_4Cu_4I_4(IN)_8(mdea)_8$

Copper iodide (60 mg, 0.32 mmol) and n-propanol (2.5 ml) were added to the reaction system of **AlOC-131** at 120°C for 4 days. When cooled to room temperature, yellow needle-like crystals were obtained.

2.3 Assembly of Al-Ln heterometallic molecular rings using elongated ligands

Synthesis of $Al_4Eu_4Cu_4I_4(pyba)_8(mdea)_8$ (AlOC-133).

A mixture of aluminum isopropoxide (102 mg, 1 mmol), 4-(4-pyridy)benzoic acid (200 mg, 1 mmol), europium nitrate hexahydrate (210 mg, 0.50 mmol), cuprous iodide (60 mg, 0.32 mmol), N-methyldiethanolamine (2.5 mL), DMF (4 mL), n-propanol (2.5 mL) and methylamine ethanol solution (40 %, 120 μL) was sealed in a 20 mL vial and transferred to a preheated oven at 100 °C for 4 days. When cooled to room temperature, yellow bulk crystals were obtained. Among them, europium nitrate hexahydrate can be replaced by samarium nitrate hexahydrate, gadolinium nitrate hexahydrate, terbium nitrate hexahydrate, dysprosium nitrate hexahydrate, holmium nitrate pentahydrate, erbium nitrate pentahydrate, thulium nitrate pentahydrate, yttrium nitrate hexahydrate and lutetium nitrate hexahydrate to obtain colorless, pink and pale yellow crystals, respectively. The crystals are rinsed with DMF and preserved under a sealed and dry environment. (yield: 65% based on Al(O^{i}Pr)$_3$). FT-IR (KBr, cm^{-1}): 3067(w), 2960(w), 2851(m), 2799(w), 2701(w), 1667(s), 1588(s), 1555(s), 1394(s), 1244(w), 1089(s).**3. Supplementary Figures**.

3.1 Syntheses.

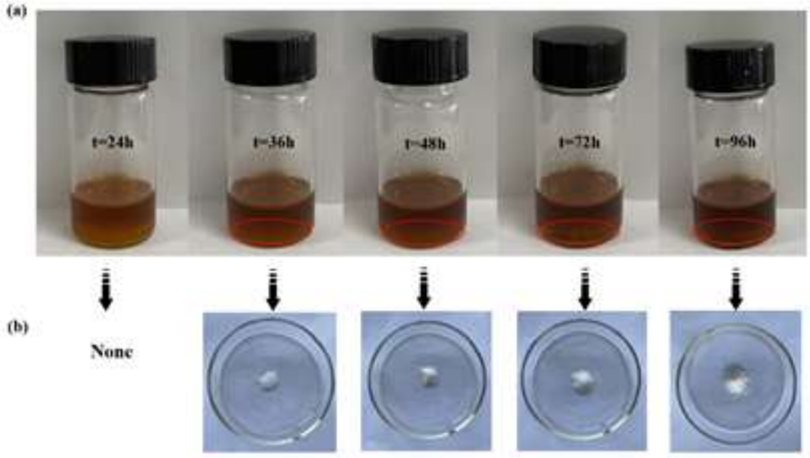

Supplementary Figure 1. Pictures of (a) reactor and (b) crystals at different time periods during the reaction of compound **AlOC-130** at a temperature of 120 °C.

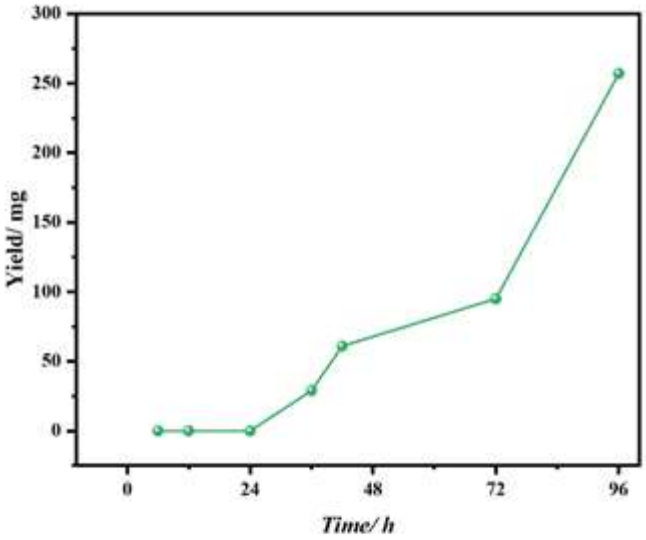

Supplementary Figure 2. Plot of reaction time versus yield for the compound **AlOC-130** at a reaction temperature of 120°C.

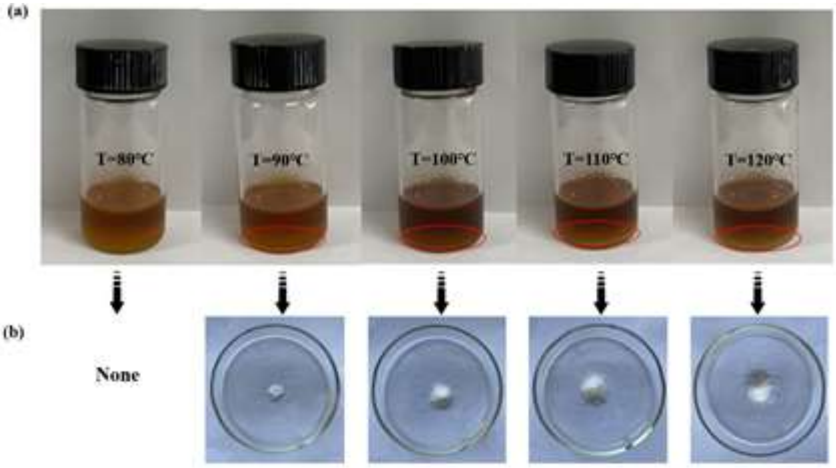

Supplementary Figure 3. Photos of (a) reactor and (b) crystals of compound **AlOC-130** at different temperatures after 96 h of reaction time.

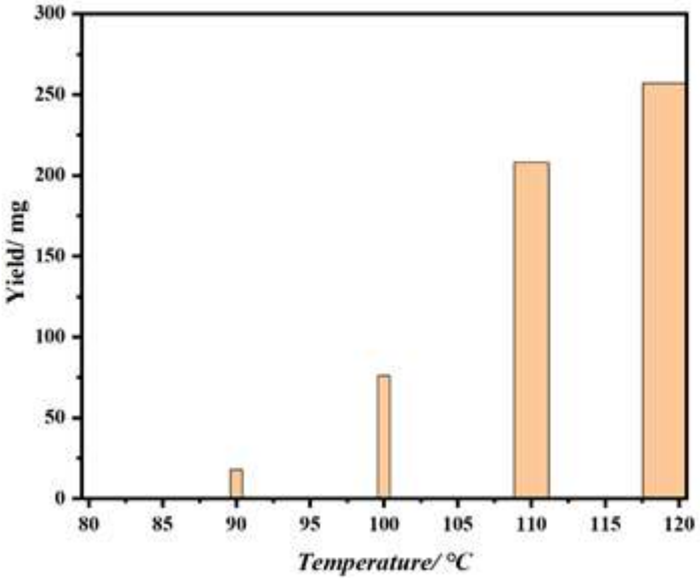

Supplementary Figure 4. Plot of temperature versus yield for compound **AlOC-130** at reaction time of 96h.

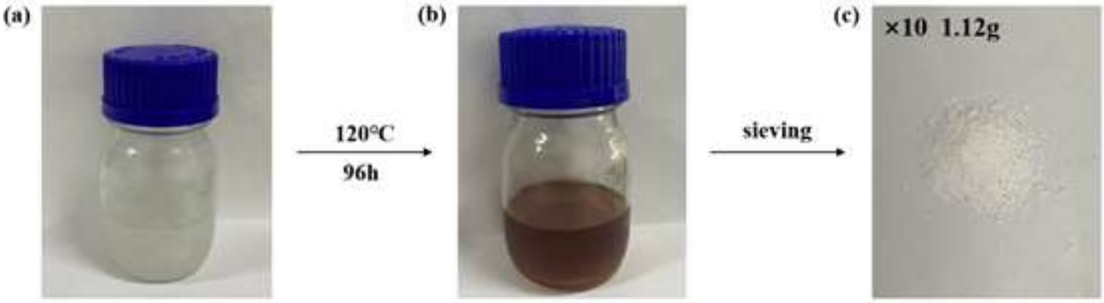

Supplementary Figure 5. Scale-up synthesis of **AlOC-131**. (a) Picture of the reactor after sonication (b) Picture of the reactor after completion of the reaction, (c). Photograph of crystals after sieving with a 200 molybdenum sieve.

3.2 PXRD analysis for heterometallic compounds.

The experimental PXRD patterns for **AlOC-130** to **AlOC-133** are consistent with the simulated ones from SCXRD, which indicates that the samples are pure (Supplementary Figures 6–9). The differences in intensity between the experimental and simulated patterns might be due to the variation in crystal orientation for power samples.

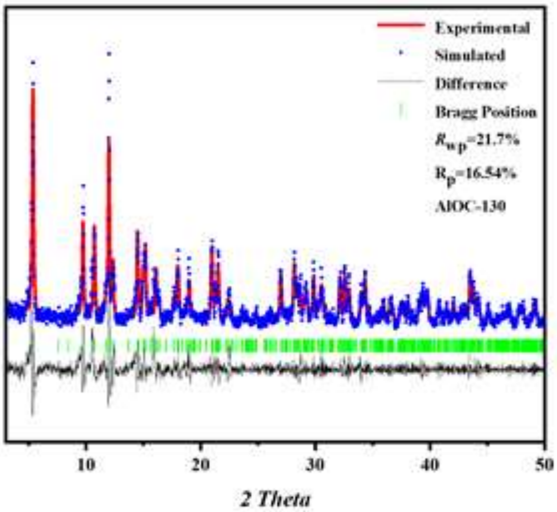

Supplementary Figure 6. Simulated PXRD patterns (black) and experimental PXRD patterns (red) of **AlOC-130**.

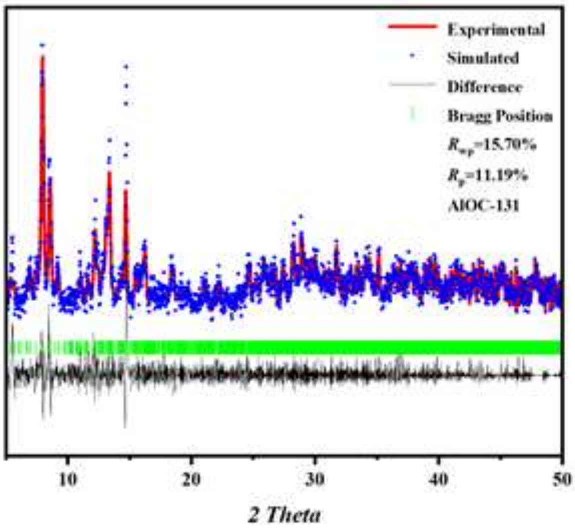

Supplementary Figure 7. Simulated PXRD patterns (black) and experimental PXRD patterns (red) of **AlOC-131**.

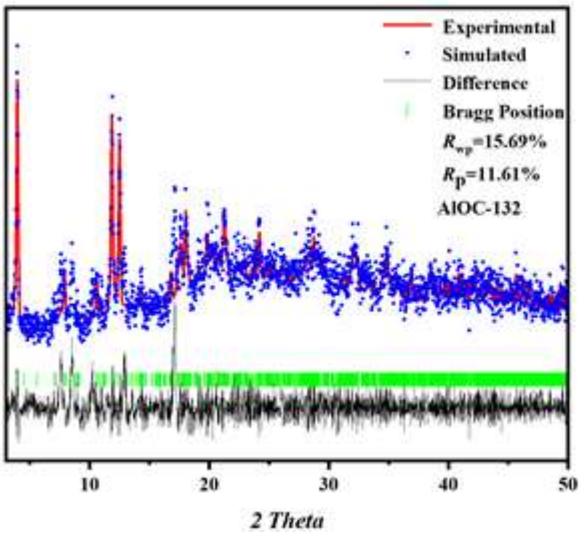

Supplementary Figure 8. Simulated PXRD patterns (black) and experimental PXRD patterns (red) of **AlOC-132**.

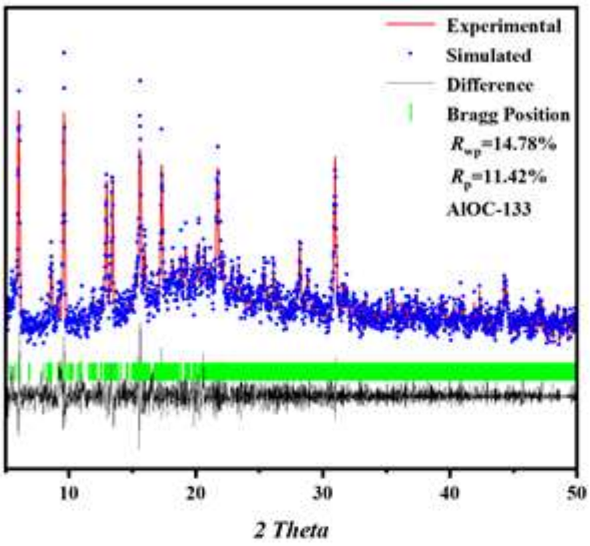

Supplementary Figure 9. Simulated PXRD patterns (black) and experimental PXRD patterns (red) of **AlOC-133**.

PXRD experiments were performed on the isostructures of **AlOC-130** to **AlOC-131** obtained by replacing different heavy rare earth ions (europium-lutetium). The experimental and simulated patterns are found to be in agreement. It shows the universality of the structure for heavy rare earth ions, and the samples are pure.

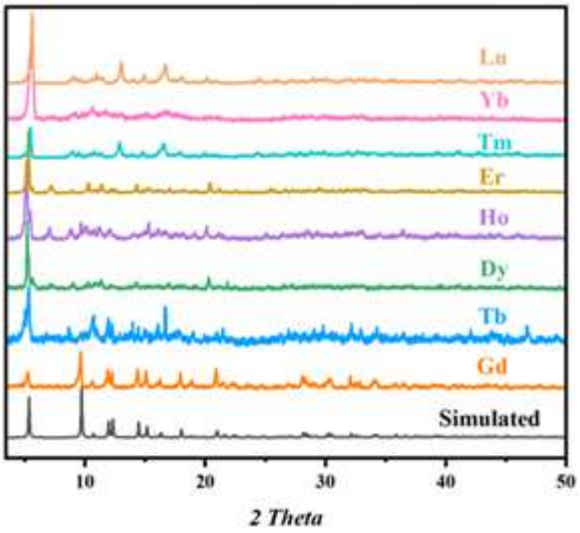

Supplementary Figure 10. Simulated and experimental PXRD patterns of **AlOC-130** replacing different rare earth elements.

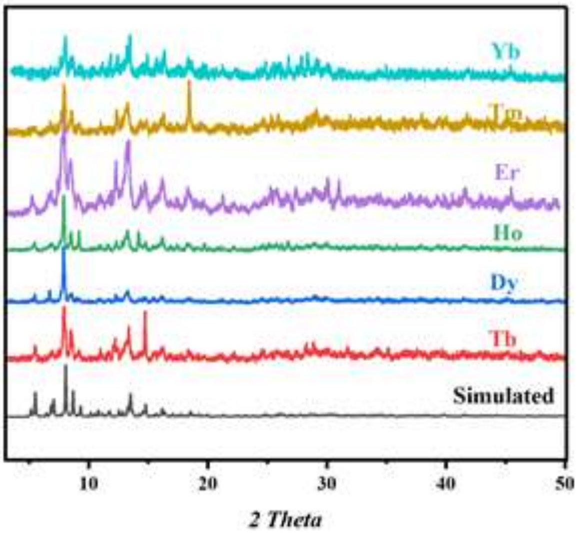

Supplementary Figure 11. Simulated and experimental PXRD patterns of **AlOC-131** replacing different rare earth elements.

3.3 Stability of heterometallic compounds.

The crystals of compounds **AlOC-130** to **AlOC-131** were immersed in organic solvents for 24h and PXRD experiments were performed. As shown in Supplementary Figures 12-13, the experimentally obtained patterns agree with the simulated ones indicating that the compounds are stable in organic solutions. And for compound **AlOC-130**, it is stable in water for 6h due to the presence of hydrophobic ligand benzoic acid, while others are unstable (Supplementary Figure 14).

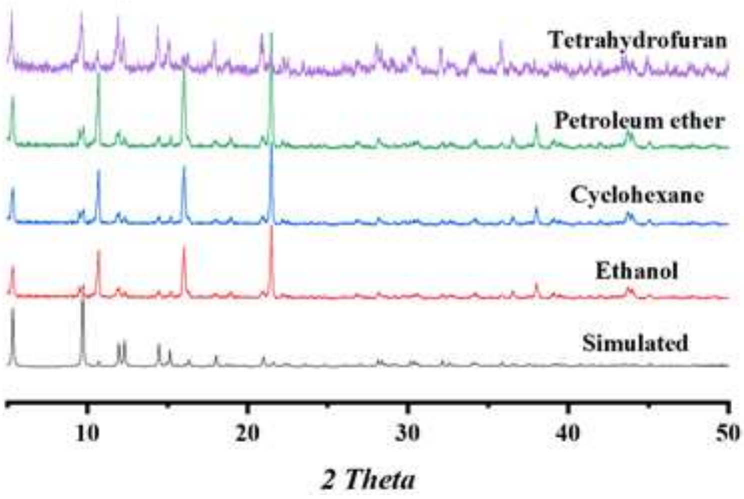

Supplementary Figure 12. PXRD patterns of **AlOC-130** in different organic solvents at room temperature for 24 h.

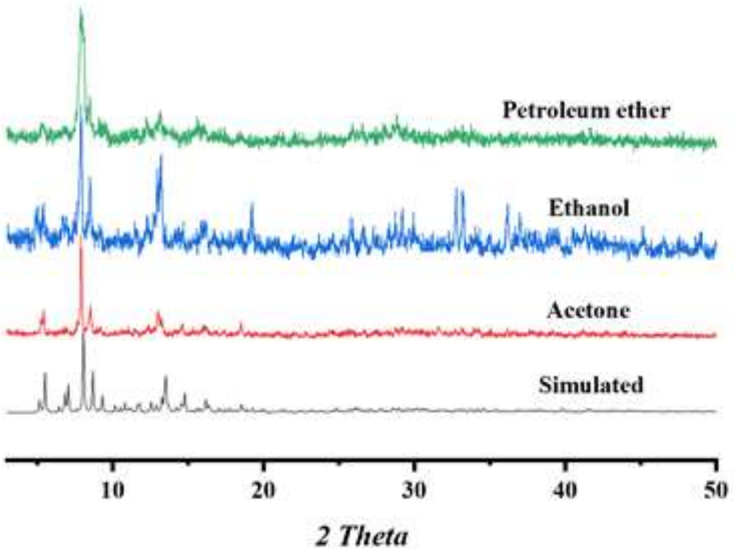

Supplementary Figure 13. PXRD patterns of **AlOC-131** in different organic solvents at room temperature for 24 h.

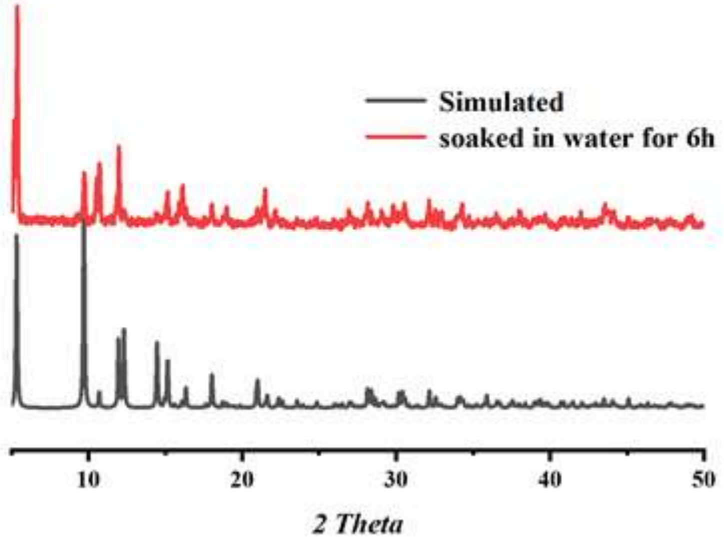

Supplementary Figure 14. PXRD patterns of **AlOC-130** in water at room temperature for 6 h.

The variable temperature PXRD of compounds **AlOC-130** to **AlOC-131** showed that they remained thermally stable until 300 °C (Supplementary Figures. 15-16).

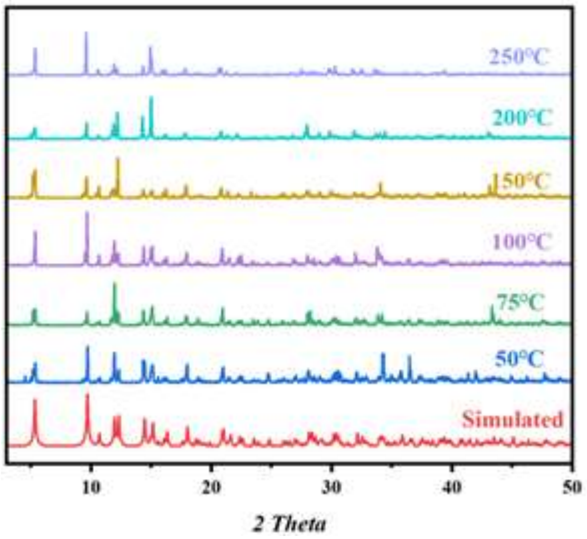

Supplementary Figure 15. The Temperature-dependent PXRD patterns of **AlOC-130**.

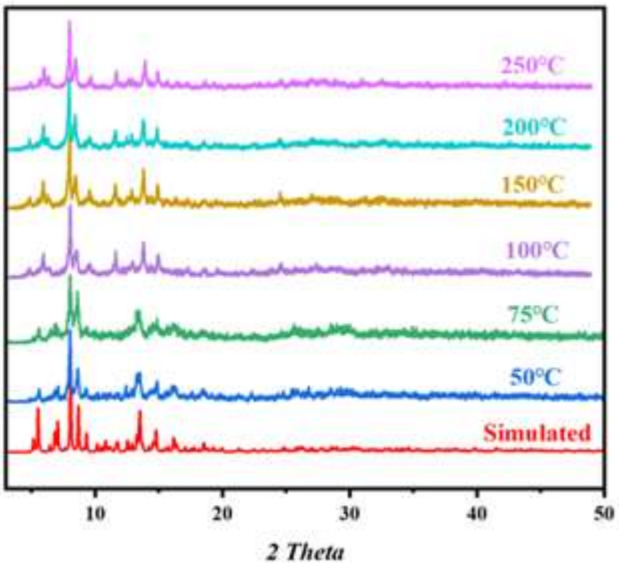

Supplementary Figure 16. The Temperature-dependent PXRD patterns of **AlOC-131**.

3.4 TGA test for heterometallic compounds.

The thermogravimetric analyses (TGA) of **AlOC-130** to **AlOC-133** show that heterometallic rings decompose at 300 –350 ^{o}C. The first weight loss of 3.26% for **AlOC-130**, 8.78% for **AlOC-131**, 4.46% for **AlOC-132**, 4.21% for **AlOC-133** corresponded to the release of guests and unresolved solvent molecules. All heterometallic molecular ring compounds show severe weight loss after 350°C indicating a collapse of the ring skeleton (Supplementary Figures 17-20).

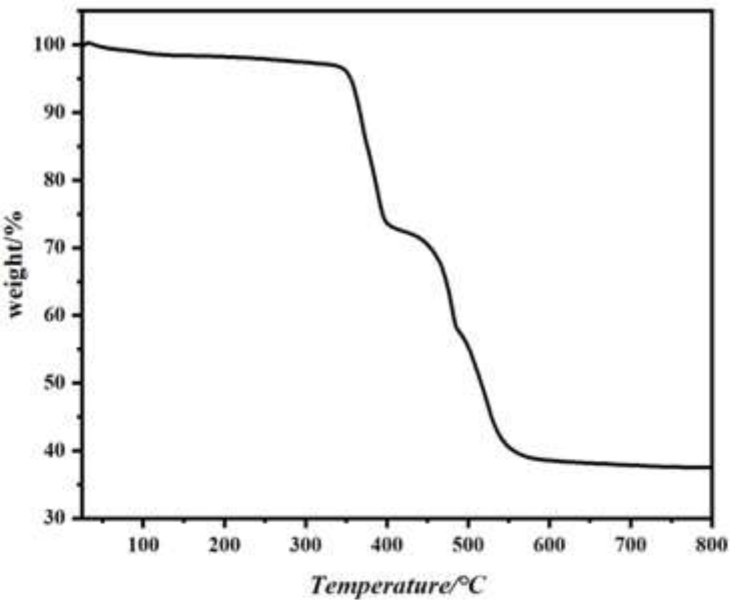

Supplementary Figure 17. The TGA curve of **AlOC-130**.

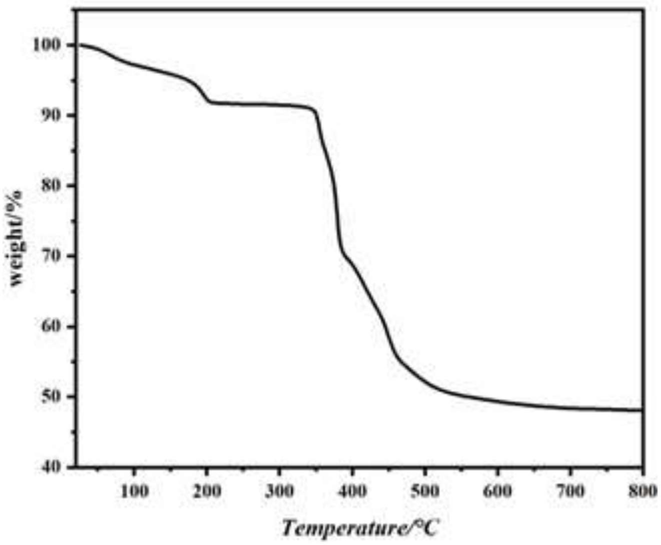

Supplementary Figure 18. The TGA curve of **AlOC-131**.

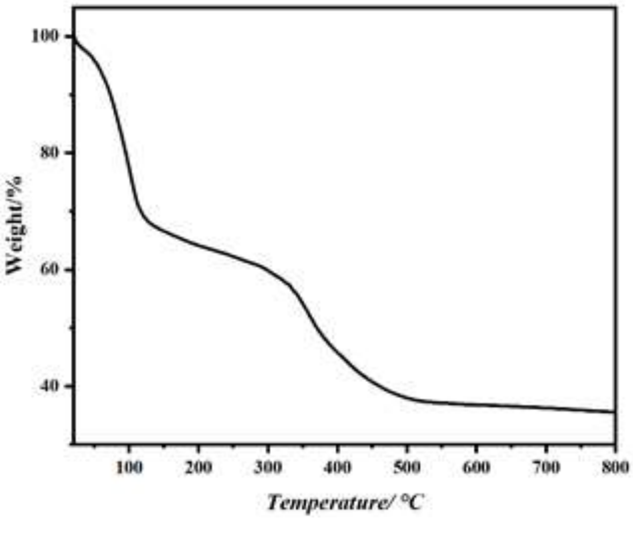

Supplementary Figure 19. The TGA curve of **AlOC-132**.

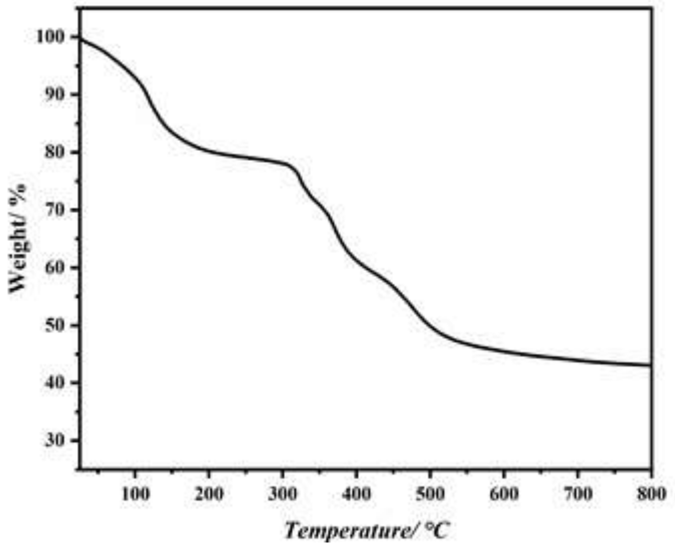

Supplementary Figure 20. The TGA curve of **AlOC-133**.

3.5 FT-IR spectra for heterometallic compounds.

The IR spectra of heterometallic rings have been recorded in the range of 4000–400 cm^{-1} from solid samples palletized with KBr, which are presented in Supplementary Figures S21-S24. The substitution of different rare earth elements in the compound AlOCs does not affect the peak position and intensity of the functional groups. The incorporation of isonicotinic acid, benzoic acid, and formic acid in AlOCs is supported by the C=O vibrations at 1667 $cm^{-1}$and 1485 cm^{-1}; 1592 $cm^{-1}$and 1408 cm^{-1}; 1572 $cm^{-1}$and 1448 cm^{-1}, respectively. The aromatic ring C-H stretching-vibrations can be seen at 3058 cm^{-1} and 3050 cm^{-1}. And the aliphatic C-H stretching-vibrations of the N-methyldiethanolamine molecular occurs at about 2963 cm^{-1} and 2880 cm^{-1}.

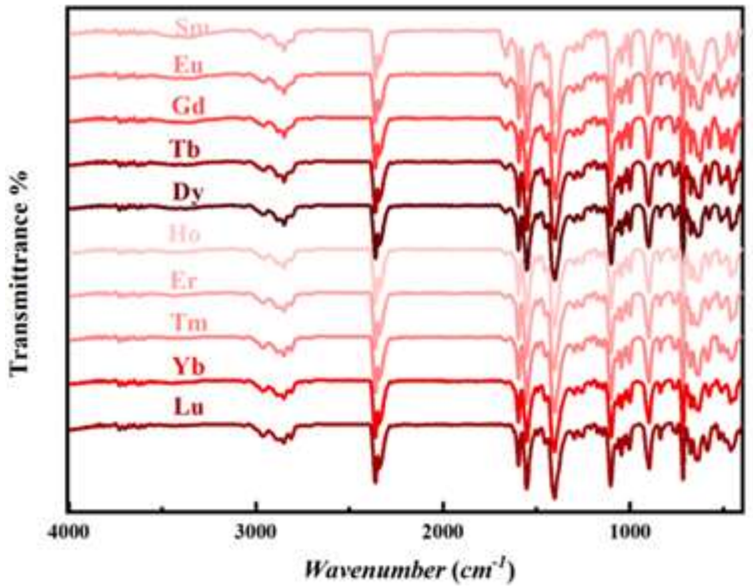

Supplementary Figure 21. IR spectrum of **AlOC-130**.

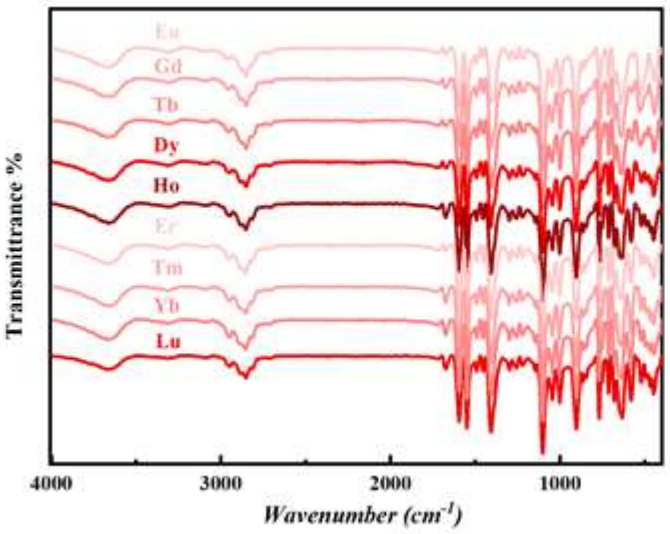

Supplementary Figure 22. IR spectrum of **AlOC-131**.

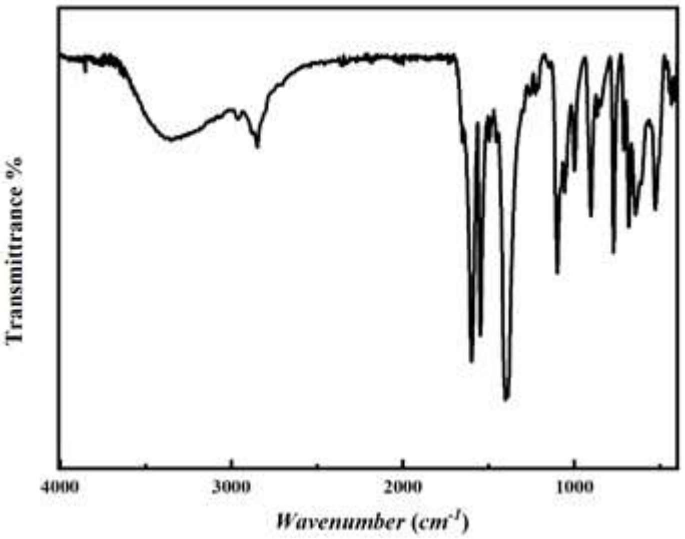

Supplementary Figure 23. IR spectrum of **AlOC-132**.

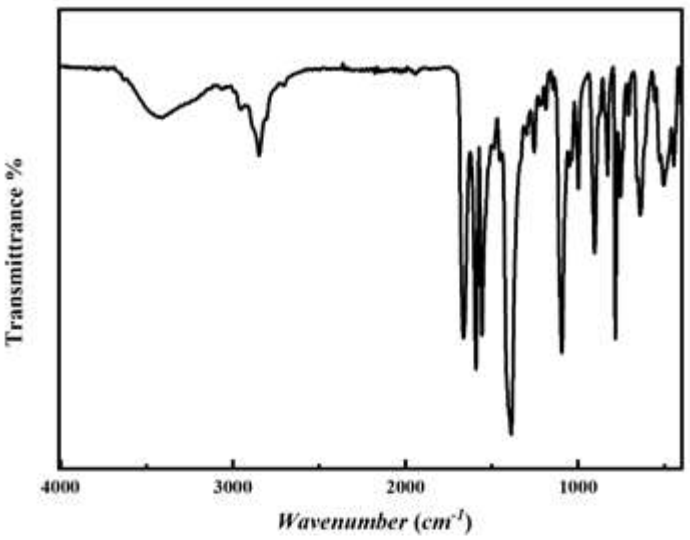

Supplementary Figure 24. IR spectrum of **AlOC-133**.

3.6 The solid-state absorption spectra of heterometallic compounds.

UV-vis spectra of colorless crystals **AlOC-130** to **AlOC-131** display narrow absorptions ranging from 300 to 350 nm, and the calculated adsorption edges are respectively 3.20 eV for **AlOC-130**, 3.40 eV for **AlOC-131**, (Supplementary Figures 25–26). Because Cu_4I_4 is connected to heterometallic rings, the compound **AlOC-132** to **AlOC-133** crystals are yellow and present enhanced adsorption from 500 to 550 nm, and the calculated adsorption edges are respectively 1.84 eV for **AlOC-132**, 1.83 eV for **AlOC-133** (Supplementary Figures 27-28).

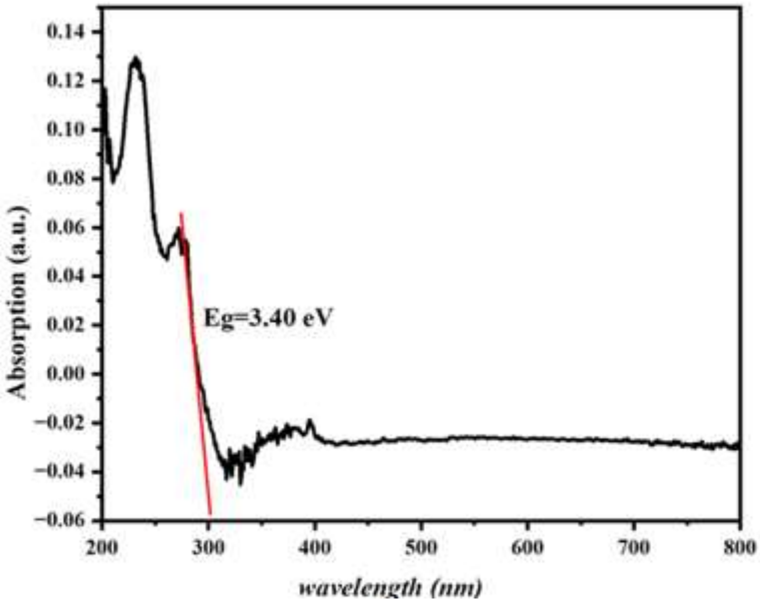

Supplementary Figure 25. The solid-state absorption spectra of **AlOC-130**.

Supplementary Figure 26. The solid-state absorption spectra of **AlOC-131**.

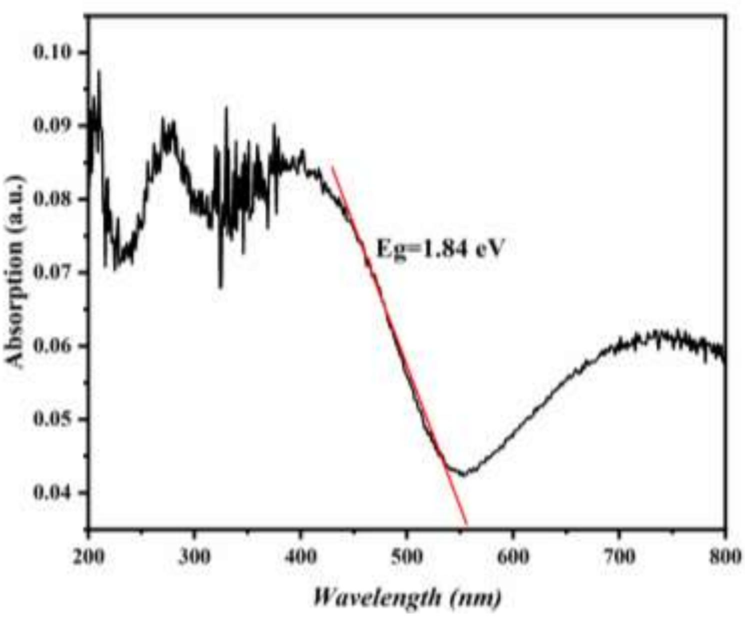

Supplementary Figure 27. The solid-state absorption spectra of **AlOC-132.**

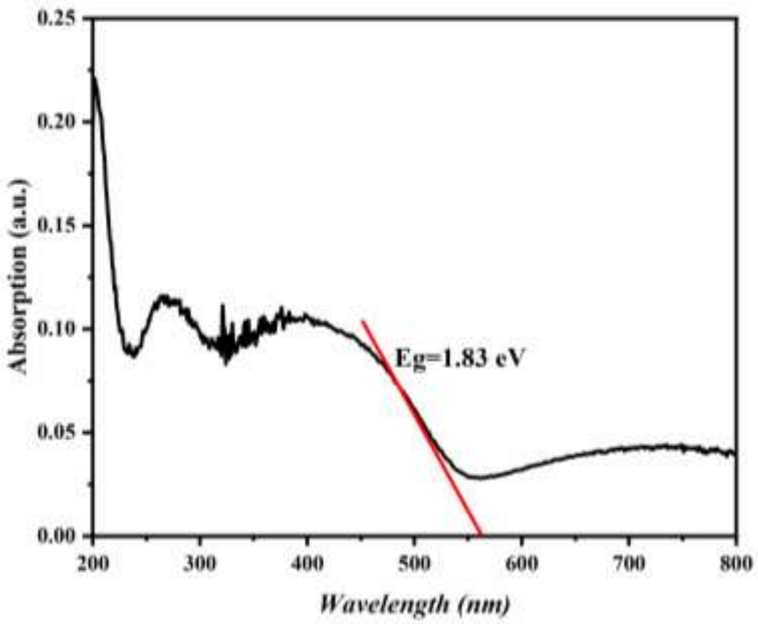

Supplementary Figure 28. The solid-state absorption spectra of **AlOC-133.**

3.7 EDS spectra for heterometallic compounds.

The presence of elements (Al, Eu, C, N and O) in **AlOC-130** to **AlOC-131** and elements (Al, Eu, Cu, I, C, N, O) in **AlOC-132** to **AlOC-133** have been validated by EDS spectra (Supplementary Figures 29–32).

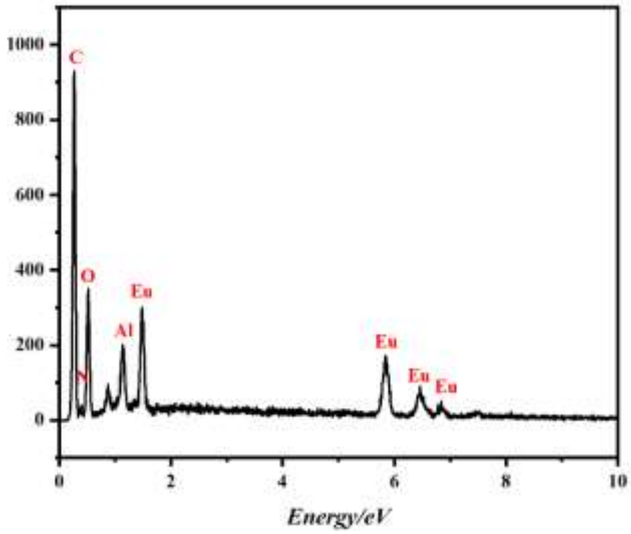

Supplementary Figure 29. The EDS spectrum of compound **AlOC-130** $Al_4Eu_4(BA)_8(mdea)_8$.

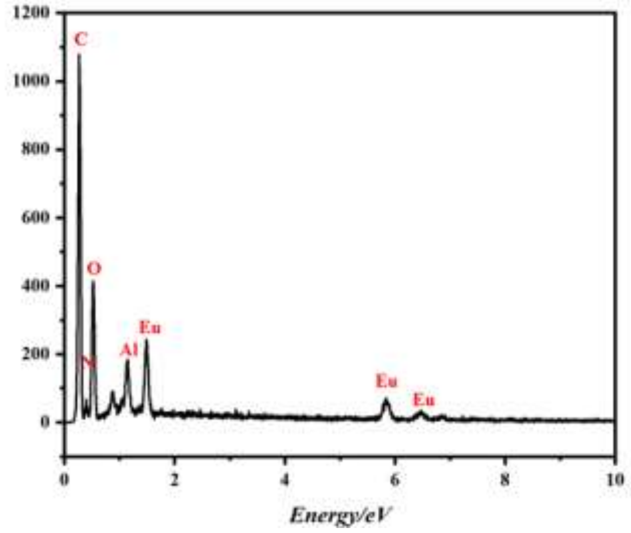

Supplementary Figure 30. The EDS spectrum of compound **AlOC-131** [$Al_4Eu_4(IN)_8(mdea)_8(H_2O)$]$\cdot 2H_2O$.

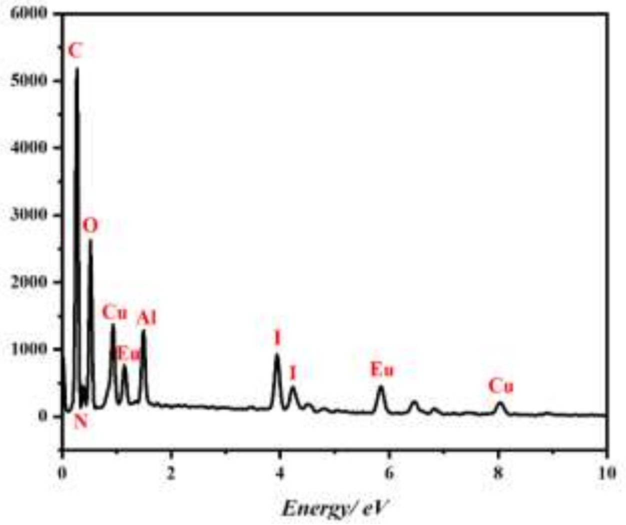

Supplementary Figure 31. The EDS spectrum of compound **AlOC-132** $Al_4Eu_4Cu_4I_4(IN)_8(mdea)_8$.

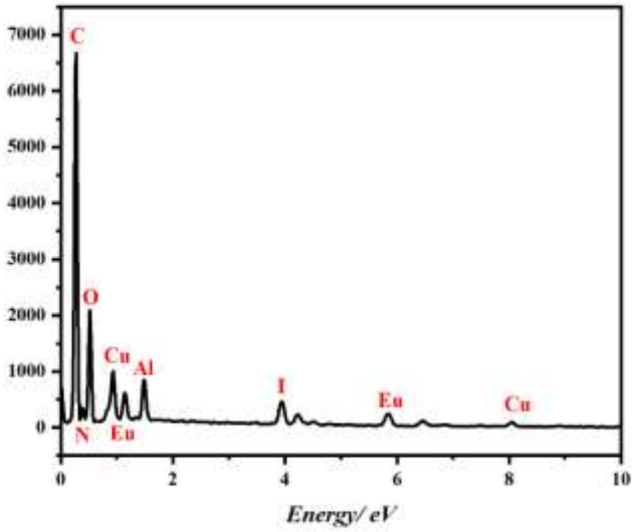

Supplementary Figure 32. The EDS spectrum of compound **AlOC-133** $Al_4Eu_4Cu_4I_4(pyba)_8(mdea)_8$.

3.8 EDS-mapping spectra for heterometallic compounds.

The distribution of elements in **AlOC-130** to **AlOC-131** (Al, Ln, C, N and O) and **AlOC-132** to **AlOC-133** (Al, Ln, Cu, C, N, O and I) was characterized by eds-mapping (Supplementary Figures 33–36).

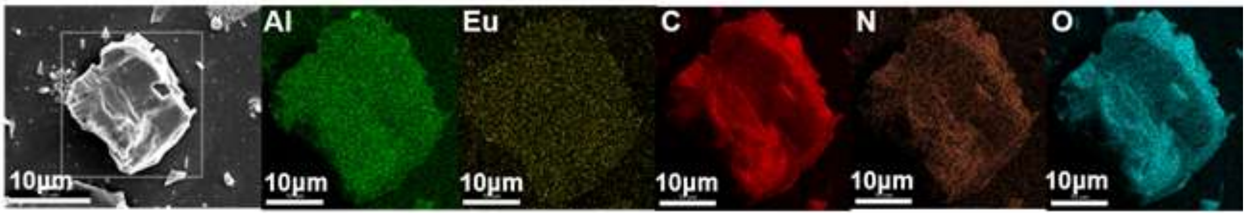

Supplementary Figure 33. EDS-mapping spectra of **AlOC-130**. From left to right are crystal appearance, Al, Ln, C, N and O elements.

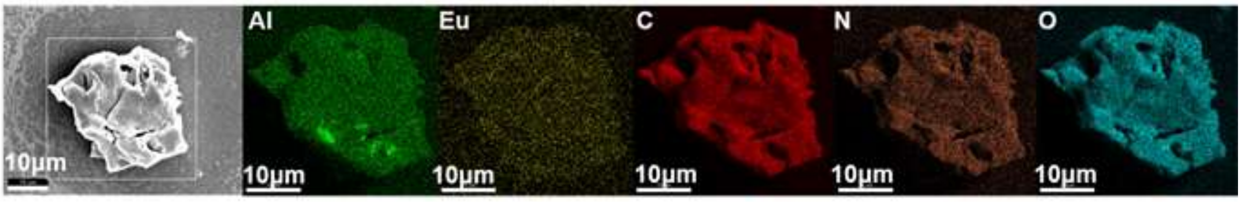

Supplementary Figure 34. EDS-mapping spectra of **AlOC-131**. From left to right are crystal appearance, Al, Ln, C, N and O elements.

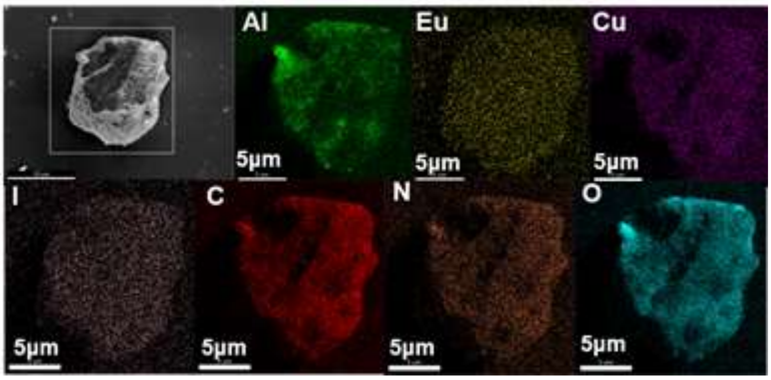

Supplementary Figure 35. EDS-mapping spectra of **AlOC-132**. From left to right are crystal appearance, Al, Ln, Cu, I and C elements.

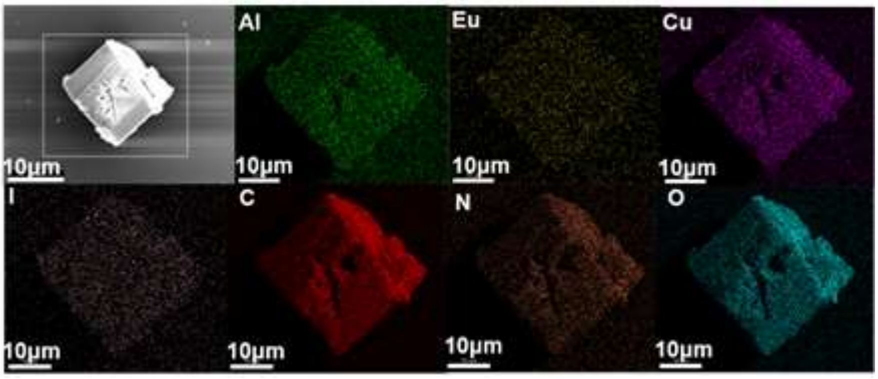

Supplementary Figure 36. EDS-mapping spectra of **AlOC-133**. From left to right are crystal appearance, Al, Ln, Cu, I and C elements.

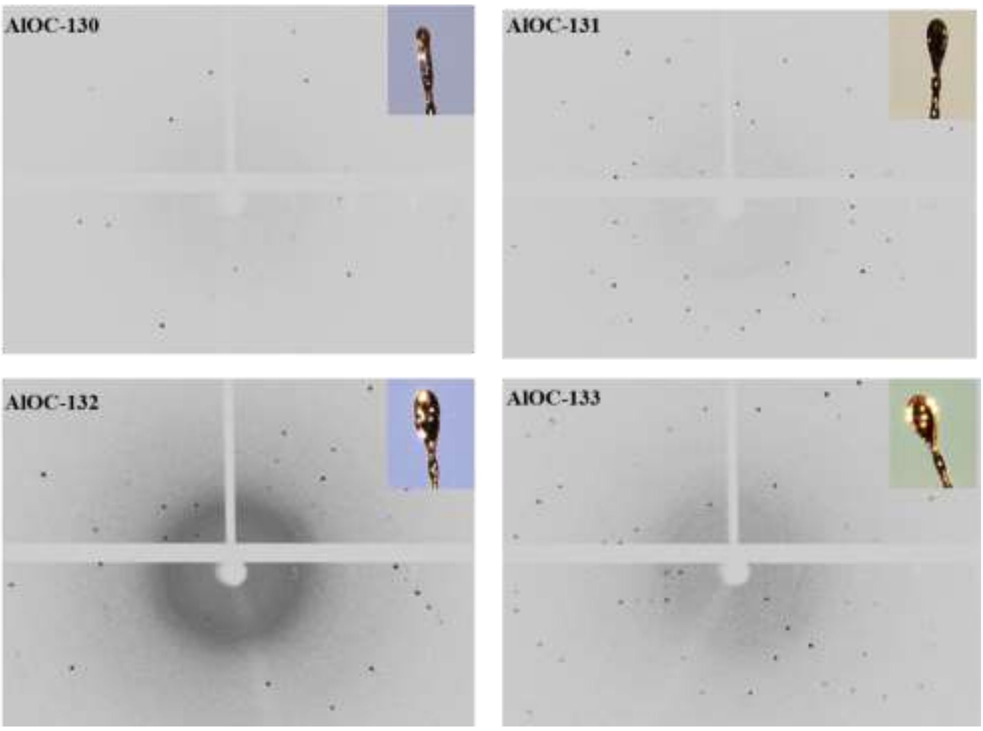

Supplementary Figure 37. Single crystal X-ray diffraction images of heterometallic rings, the inserted photographs show the color and shapes of heterometallic rings.

3.9 Detailed Structure Information for Heterometallic Rings.

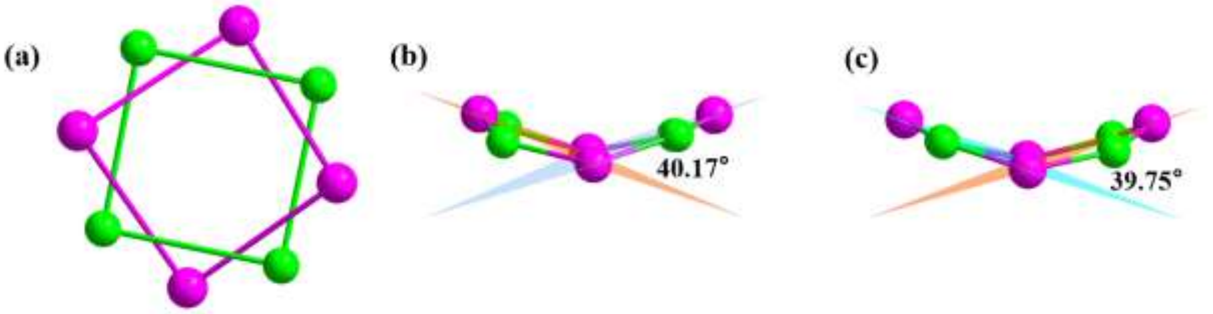

Supplementary Figure 38. (a) Composition of the inorganic $\{Al_4Ln_4\}$ unit; the heterometallic dihedral angle of (b) compound **AlOC-130**; (c) compound **AlOC-131**. Hydrogen atoms are omitted for clarity. Color code: Al, green; Eu: pink.

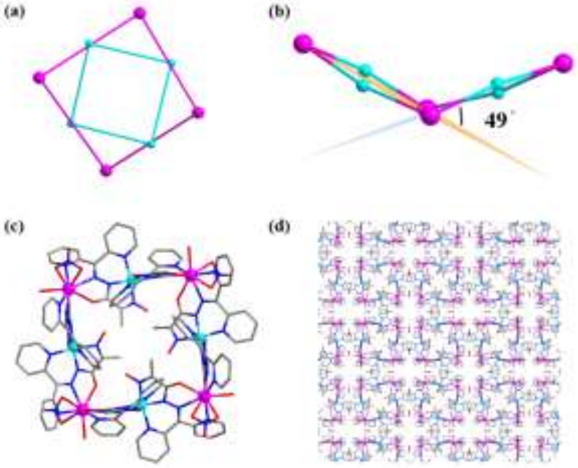

Supplementary Figure 39. (a) Composition of the inorganic {Fe_4Ln_4} unit; (b) the heterometallic dihedral angle of compound $Fe^{III}_4Ln^{III}_4$; (c) Top view and (d) stacking diagram of compound $Fe^{III}_4Ln^{III}_4$.[2] Hydrogen atoms are omitted for clarity. Color code: Fe, blue; Eu: pink; C, gray; O, red; N, blue.

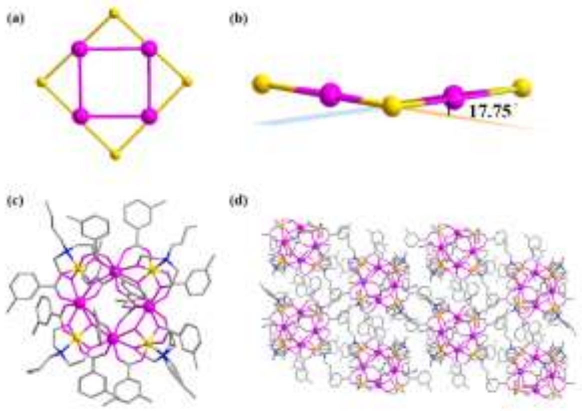

Supplementary Figure 40. (a) Composition of the inorganic {Ga_4Ln_4} unit; (b) the heterometallic dihedral angle of compound 5; (c) Top view and (d) stacking diagram of compound 5.[3] Hydrogen atoms are omitted for clarity. Color code: Ga, gold; Eu: pink; C, gray; O, red; N, blue.

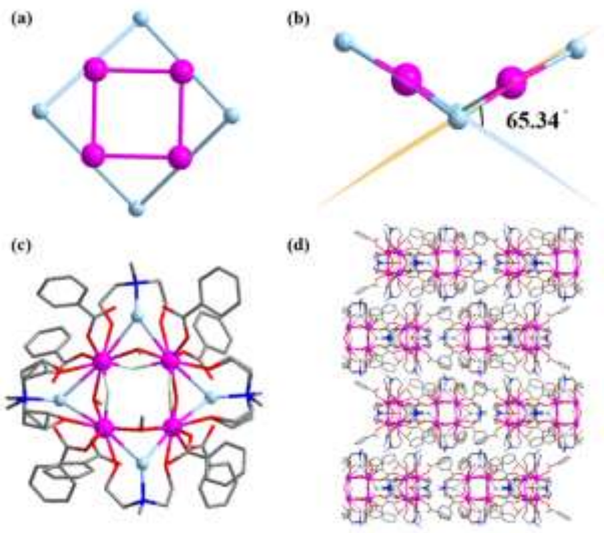

Supplementary Figure 41. (a) Composition of the inorganic {Cr_4Ln_4} unit; (b) the heterometallic dihedral angle of compound $Cr^{III}_4Ln^{III}_4$; (c) Top view and (d) stacking diagram of compound $Cr^{III}_4Ln^{III}_4$.[4] Hydrogen atoms are omitted for clarity. Color code: Cr, blue; Eu: pink; C, gray; O, red; N, blue.

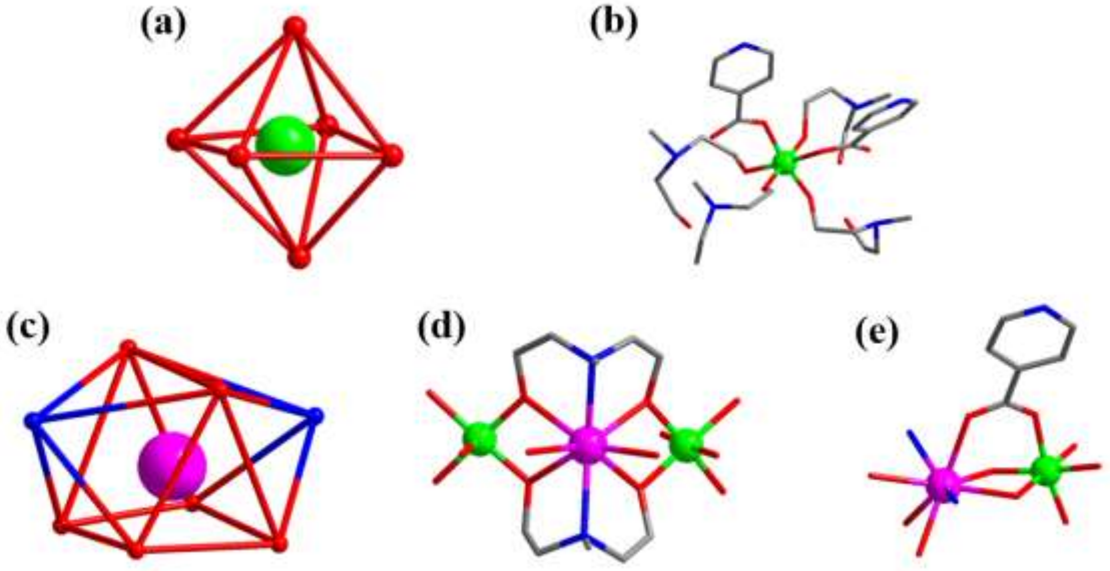

Supplementary Figure 42. (a) Al^{3+} octahedral coordination conformation; (b) Al^{3+} coordination composition; (c) Eu^{3+} coordination conformation; (d, e) Eu^{3+} coordination composition. Hydrogen atoms are omitted for clarity. Color code: Al, green; Eu: pink; C, gray; O, red; N, blue.

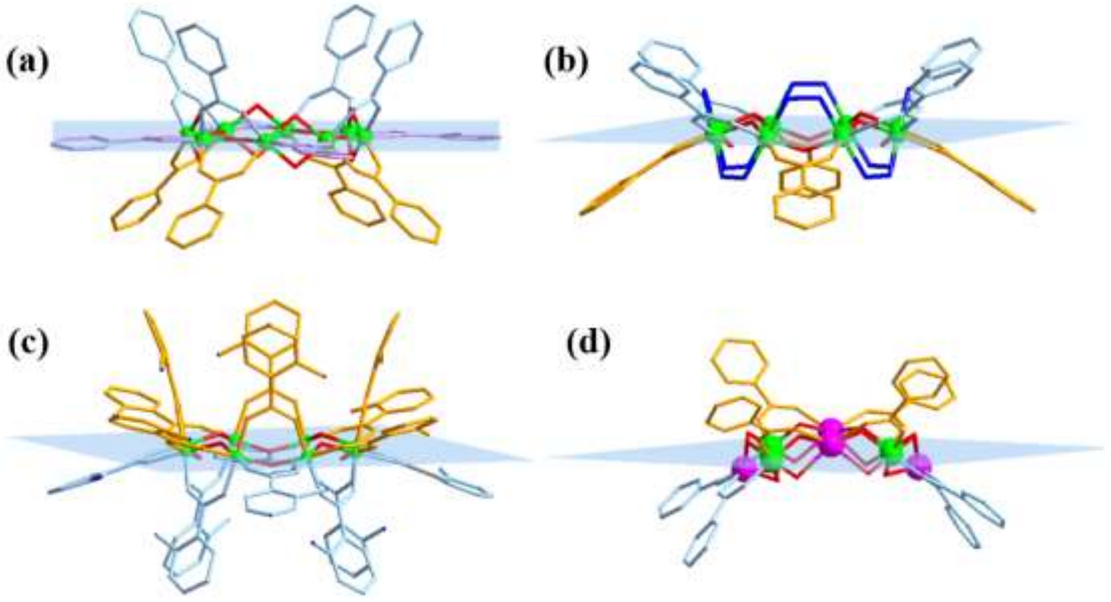

Supplementary Figure 43. Distribution of organic ligands in the plane of the molecular ring. (a) In compound AlOC-1, the organic ligands between adjacent Al^{3+} alternately appear distributed above, below and inside the aluminum ring plane.[5] (b) In compound AlOC-27, adjacent organic ligands are distributed above and below the aluminum ring plane, respectively.[6] (c) In compound AlOC-59NT, the organic ligands between adjacent Al^{3+} are distributed above and below the aluminum ring plane.[7] (d) In compound **AlOC-130**, each of the two organic ligands as a group is distributed alternately above and below the heterometallic plane (this work). Orange organic ligands represent those located above the plane of the molecular ring, and blue those below it. Hydrogen atoms and some carbon atoms are omitted for clarity. Color code: Al, green; Eu: pink; C, gray; O, red; N, blue.

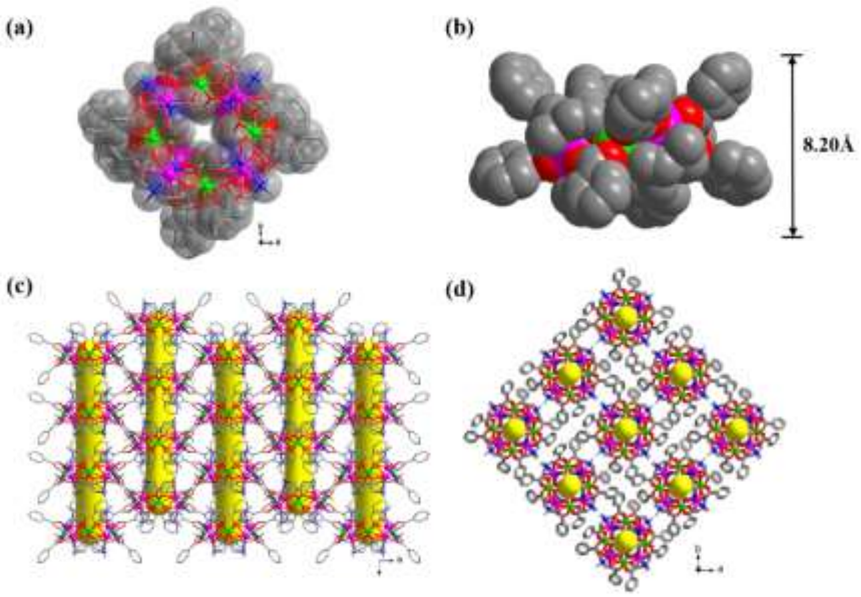

Supplementary Figure 44. Molecular structural of compound **AlOC-130** (a) top view; (b) side view; packing view along (c) [010]; (d) [001] direction. Hydrogen atoms are omitted for clarity. Color code: Al, green; Eu: pink; C, gray; O, red; N, blue.

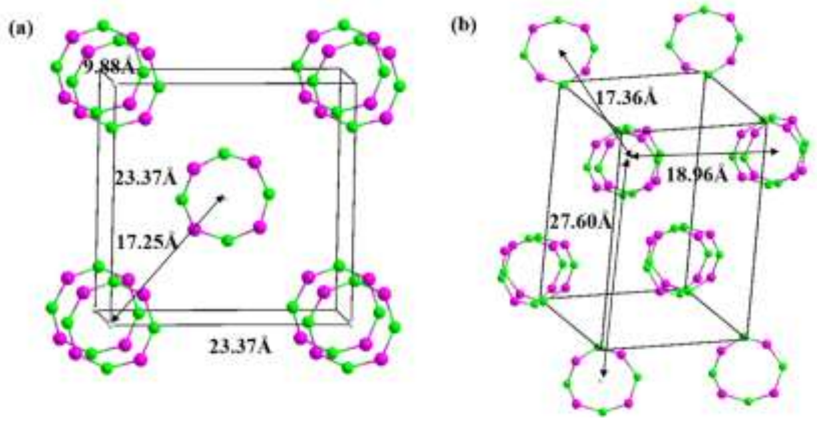

Supplementary Figure 45. Distribution of heterometallic rings and distances between them in unit cells of the compound **AlOC-130** (left) and **AlOC-131** (right). Hydrogen atoms and non-metal atoms are omitted for clarity. Color code: Al, green; Eu: pink; C, gray; O, red; N, blue.

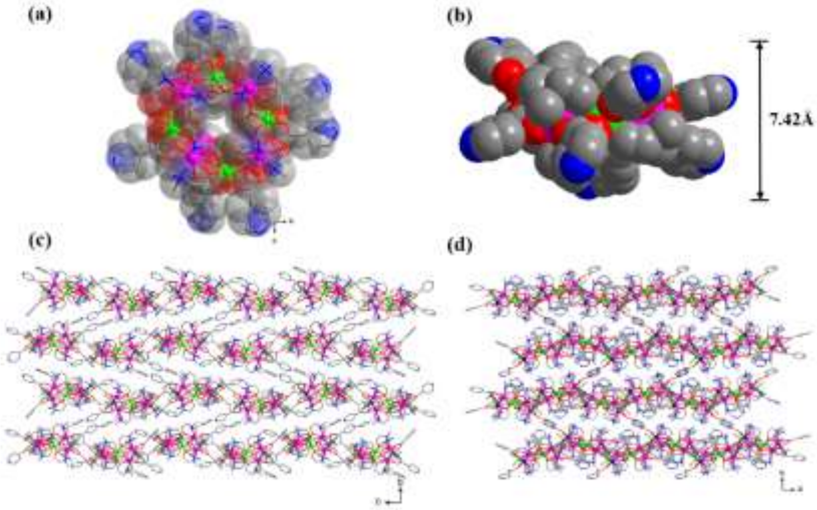

Supplementary Figure 46. Molecular structural of compound **AlOC-131** (a) top view; (b) side view; packing view along (c) [100]; (d) [001] direction. Hydrogen atoms are omitted for clarity. Color code: Al, green; Eu: pink; C, gray; O, red; N, blue.

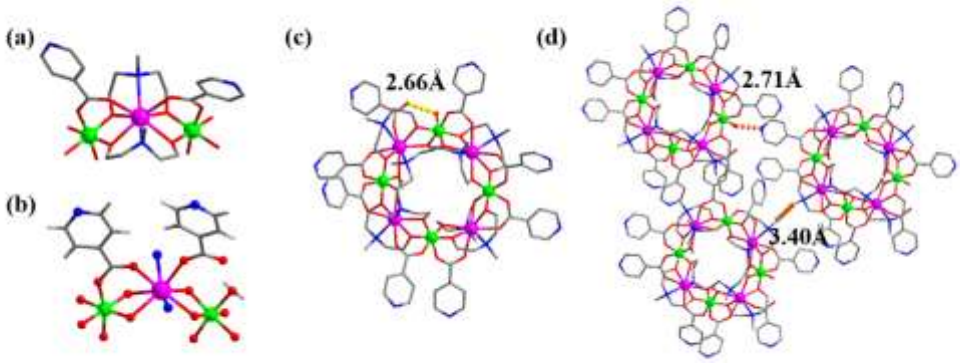

Supplementary Figure 47. Structural diagram of compound **AlOC-131** (a) coordination mode of chelating ligand; (b) coordination mode of isonicotinic acid with deletion; (c) intra-molecular ring O-H···O hydrogen bonding; (d) inter-molecular ring O-H···N, C-H···C hydrogen bonding. Hydrogen atoms are omitted for clarity. Color code: Al, green; Eu: pink; C, gray; O, red; N, blue.

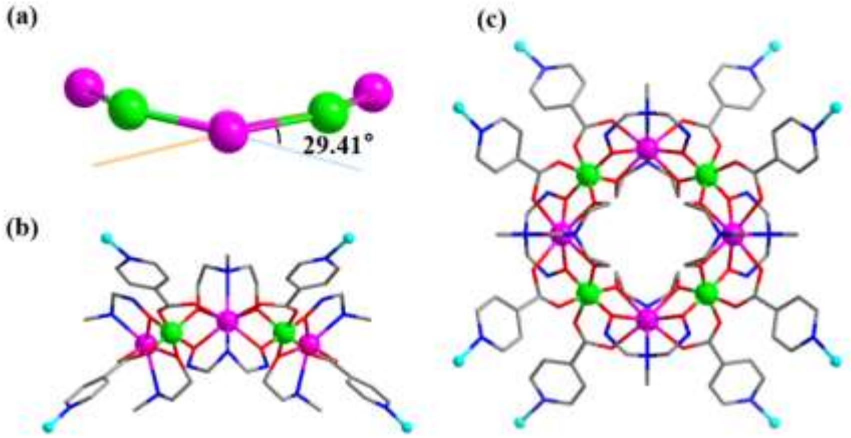

Supplementary Figure 48. (a) Heterometallic dihedral angles in $\{Al_4Ln_4\}$ unit. (c) Side view and (d) top view of 8-connected $\{Al_4Ln_4\}$ heterometallic ring in compound **AlOC-132**. Hydrogen atoms are omitted for clarity. Color code: Al, green; Eu: pink; Cu: blue; C, gray; O, red; N, blue.

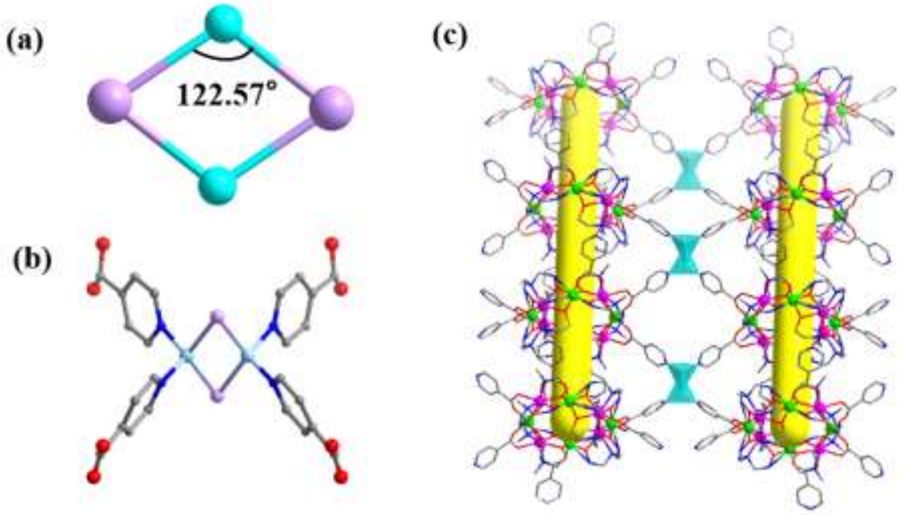

Supplementary Figure 49. (a) I-Cu-I dihedral angles in $Cu^{I}_{2}I_{2}$ linker. (b) 4-connected $Cu^{I}_{2}I_{2}$ section. (c) Coordination environment of the $Cu^{I}_{2}I_{2}$ part in the compound **AlOC-132**. Hydrogen atoms are omitted for clarity. Color code: Al, green; Eu: pink; Cu: blue; C, gray; O, red; N, blue.

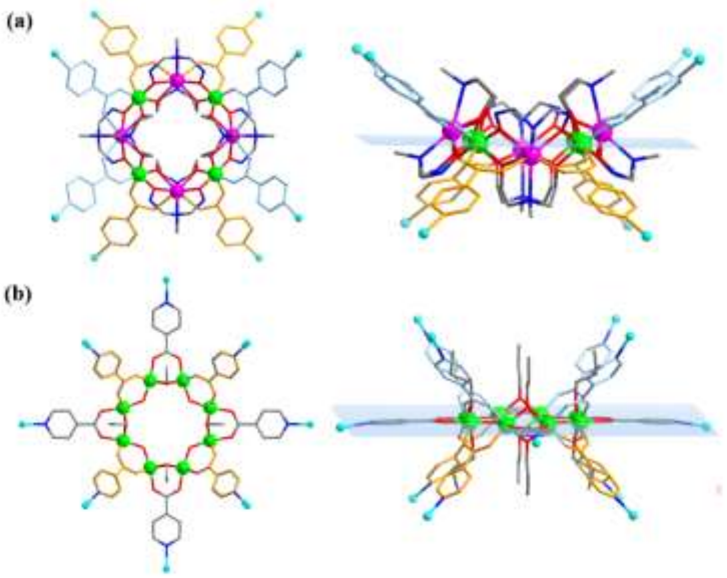

Supplementary Figure 50. (a) Top (left) and side (right) views of the $\{Al_4Ln_4\}$ unit of compound **AlOC-132**. (b) Top (left) and side (right) views of the $\{Al_8\}$ unit of compound AlOC-83.

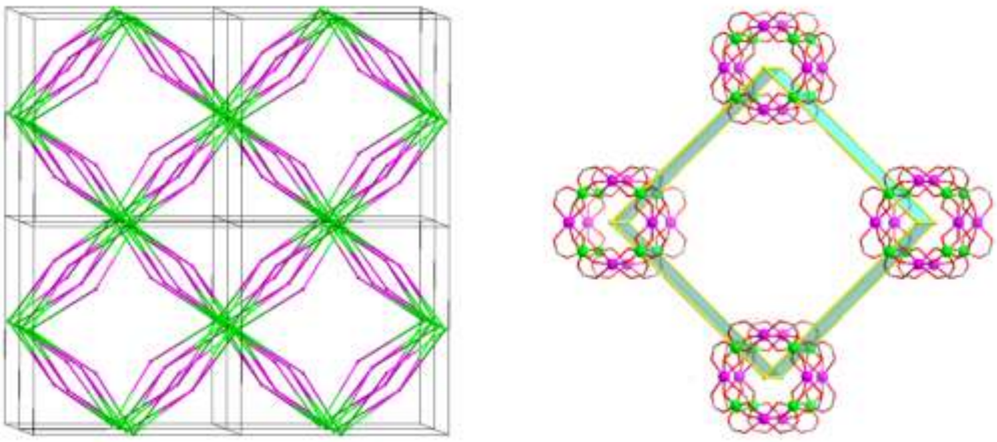

Supplementary Figure 51. The topology with {4^16.6^12} {4^4.6^2}2 points symbol of **AlOC-132**. Hydrogen atoms and non-metal atoms are omitted for clarity. Color code: Al, green; Eu: pink; Cu: blue; C, gray; O, red; N, blue.

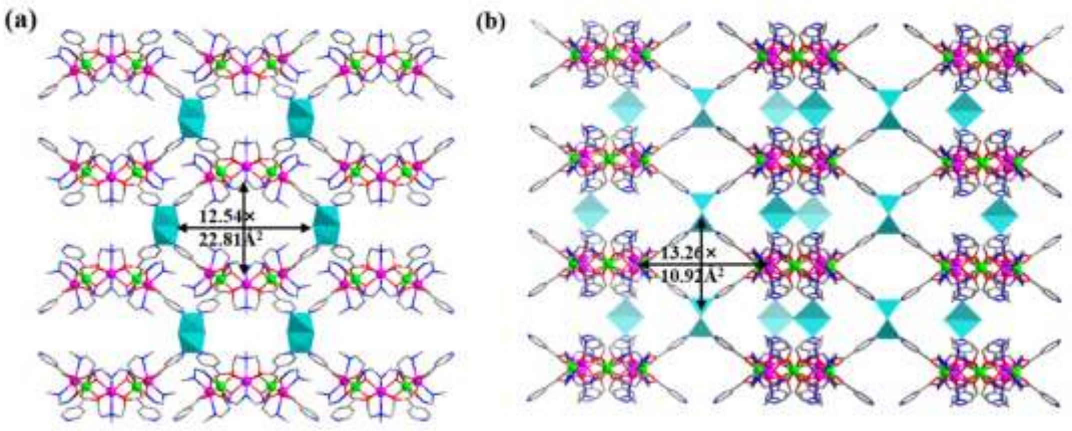

Supplementary Figure 52. Stacking diagram of compound **AlOC-132** along the (a) [100] and (b) [010] direction. Hydrogen atoms are omitted for clarity. Color code: Al, green; Eu: pink; Cu: blue; C, gray; O, red; N, blue.

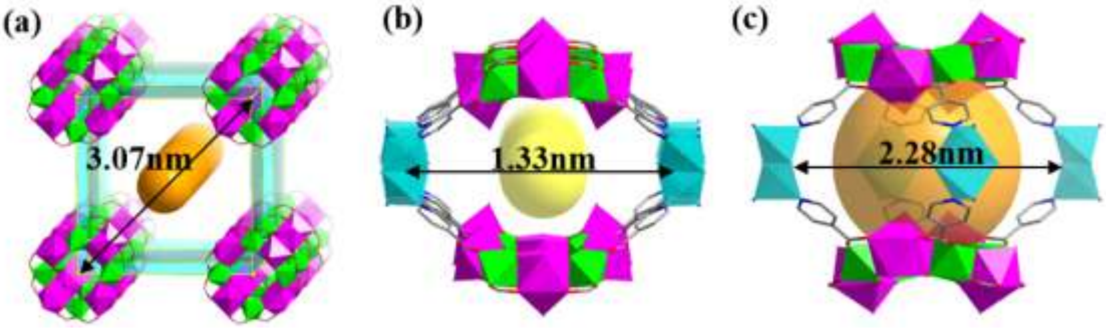

Supplementary Figure 53. (a) Type I cavity consisting of two heterometallic rings, four $[Cu^{I}_2(IN)_4I_2]$ portions and eight isonicotinic acid ligands (size: 12.54*22.81*22.81Å^3). (b) Type II cavity is composed of two heterometallic rings, two $[Cu^{I}_2(IN)_4I_2]$ parts and four isonicotinic acids in compound **AlOC-132** (size: 13.26*10.92 Å^2). Hydrogen atoms are omitted for clarity. Color code: Al, green; Eu: pink; Cu: blue; C, gray; O, red; N, blue.

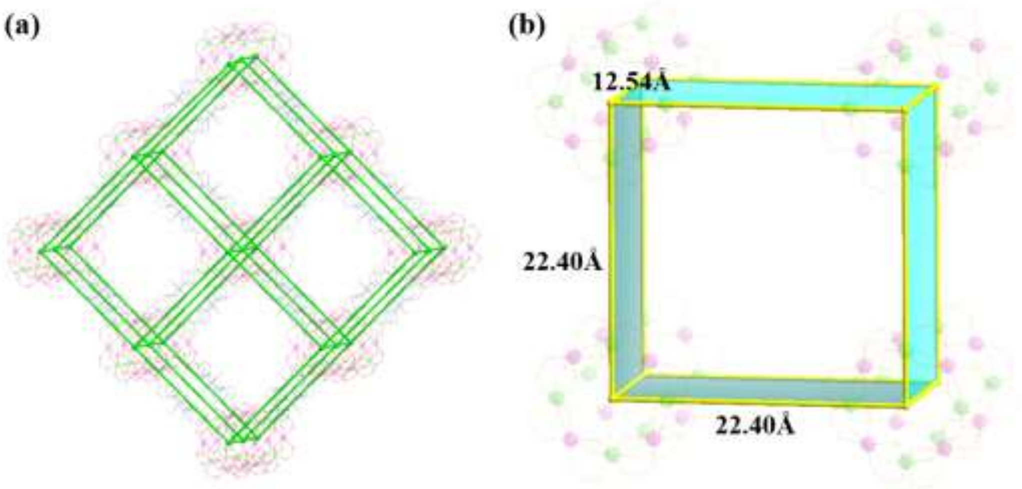

Supplementary Figure 54. (a) Distribution of heterometallic rings in the 2 × 2 × 2 cell of compound **AlOC-132**. (b) Distance between the heterometallic rings in compound **AlOC-132**. Hydrogen atoms and non-metal atoms are omitted for clarity. Color code: Al, green; Eu: pink; Cu: blue; C, gray; O, red; N, blue.

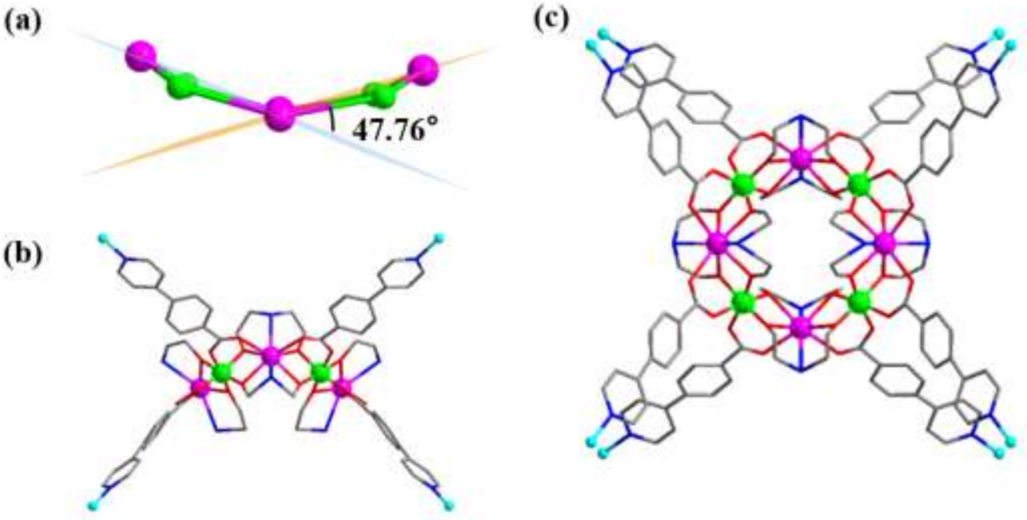

Supplementary Figure 55. (a) Heterometallic dihedral angles in {Al_4Ln_4 unit. (c) Side view and (d) top view of 8-connected {Al_4Ln_4} heterometallic ring in compound **AlOC-133**. Hydrogen atoms are omitted for clarity. Color code: Al, green; Eu: pink; Cu: blue; C, gray; O, red; N, blue.

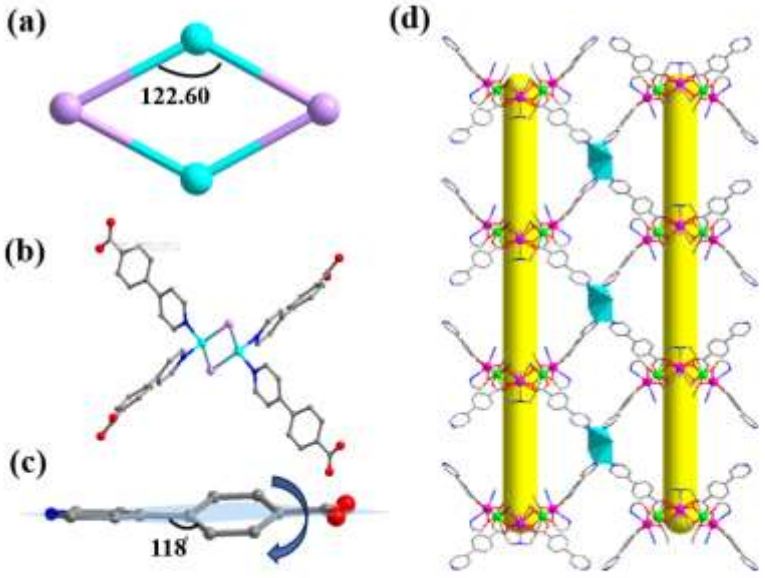

Supplementary Figure 56. (a) I-Cu-I dihedral angles in $Cu^{I}_{2}I_{2}$ linker. (b) 4-connected $Cu^{I}_{2}I_{2}$ section. (c) Torsion angle of the elongated ligand in the compound **AlOC-133**; (d) Coordination environment of the $Cu^{I}_{2}I_{2}$ part in the compound **AlOC-133**. Hydrogen atoms are omitted for clarity. Color code: Al, green; Eu: pink; Cu: blue; C, gray; O, red; N, blue.

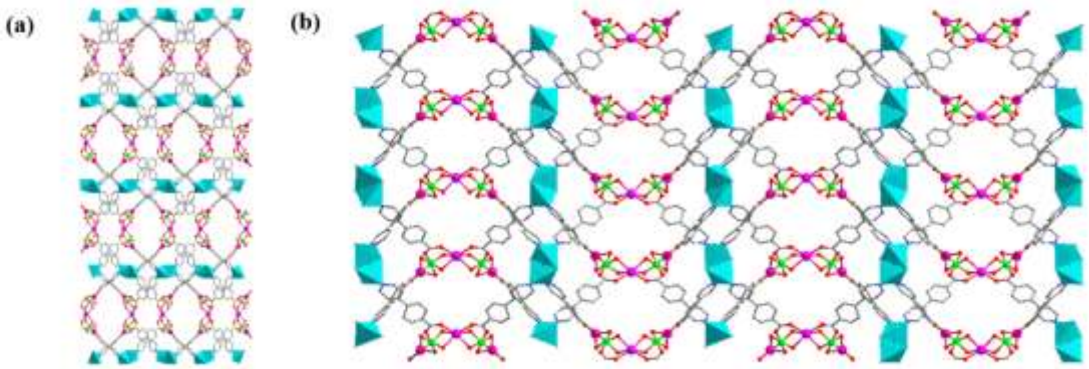

Supplementary Figure 57. Stacking diagram of compound **AlOC-133** along the (a) [100] and (b) [010] direction. Hydrogen atoms and some carbons atoms are omitted for clarity. Color code: Al, green; Eu: pink; Cu: blue; C, gray; O, red; N, blue.

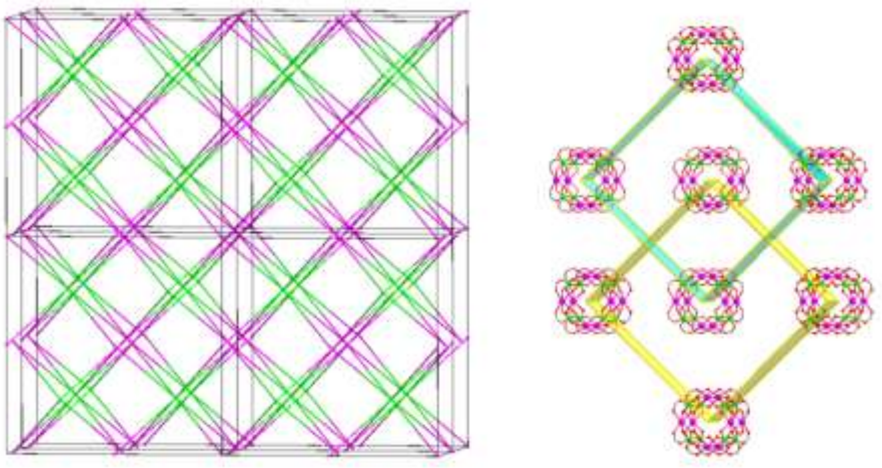

Supplementary Figure 58. The topology with {4^16.6^12} {4^4.6^2}2 points symbol of **AlOC-133**. Totally 2(1+1) interpenetrating nets. Hydrogen atoms and non-metal atoms are omitted for clarity. Color code: Al, green; Eu: pink; Cu: blue; C, gray; O, red; N, blue.

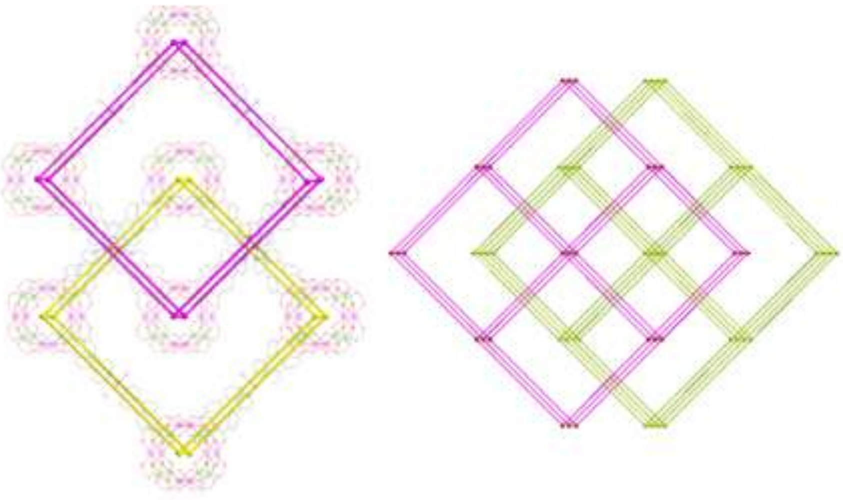

Supplementary Figure 59. Distribution of heterometallic rings in the compound **AlOC-133.** Hydrogen atoms and non-metal atoms are omitted for clarity. Color code: Al, green; Eu: pink; Cu: blue; C, gray; O, red; N, blue.

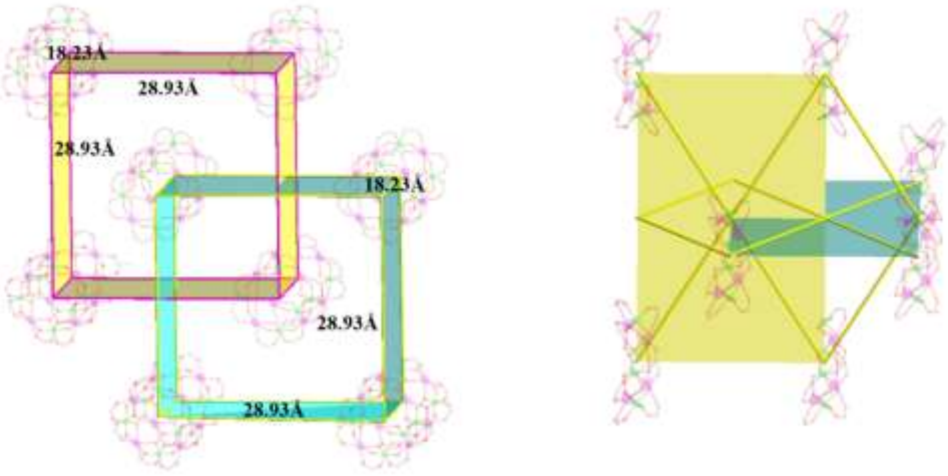

Supplementary Figure 60. Distance between the heterometallic rings in the compound **AlOC-133** and the distribution of organic ligands on the interspersed surfaces. Hydrogen atoms and non-metal atoms are omitted for clarity. Color code: Al, green; Eu: pink; Cu: blue; C, gray; O, red; N, blue. The yellow lines in the right figure represent organic ligands.

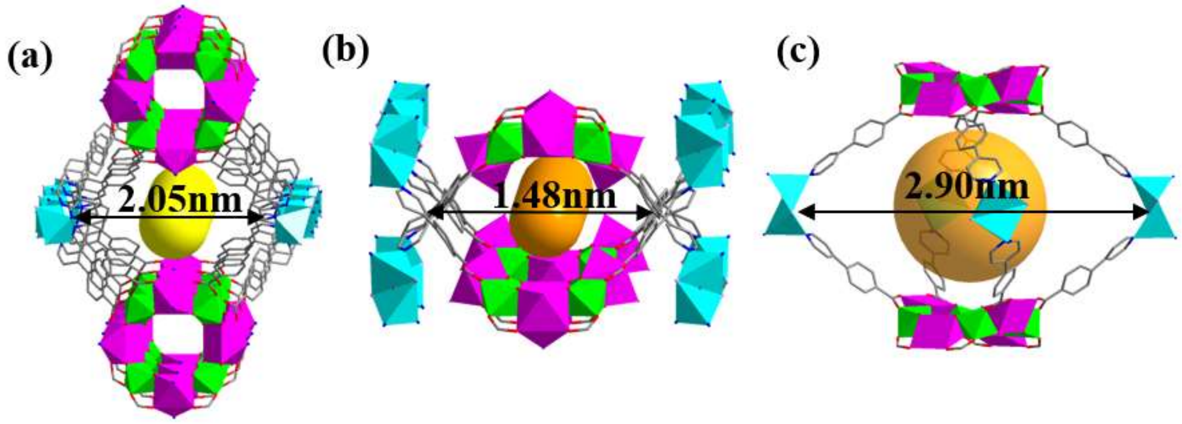

Supplementary Figure 61. (a) Type I cavity consisting of two heterometallic rings, two $[Cu^{I}_2(IN)_4I_2]$ portions and four isonicotinic acid ligands (size: 20.48*20.44Å^2). (b) Type II cavity is composed of two heterometallic rings, two $[Cu^{I}_2(IN)_4I_2]$ parts and four isonicotinic acids in compound **AlOC-133** (size: 20.48*20.44 Å^2). Hydrogen atoms are omitted for clarity. Color code: Al, green; Eu: pink; Cu: blue; C, gray; O, red; N, blue.

3.10 Heterogeneous catalytic activity of heterometallic compounds.

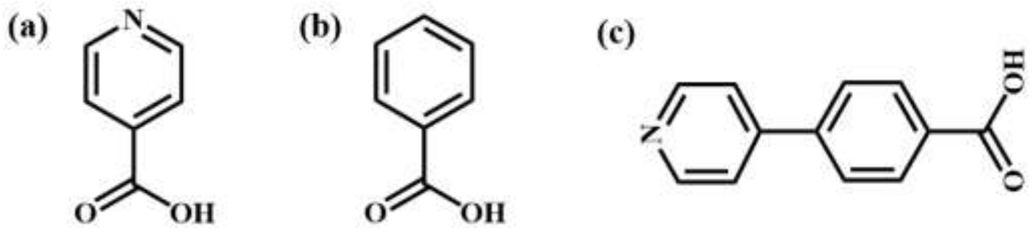

Supplementary Figure 62. Organic ligands present in the structure. (a) HIN; (b) benzoic acid; (c) Hpyba.

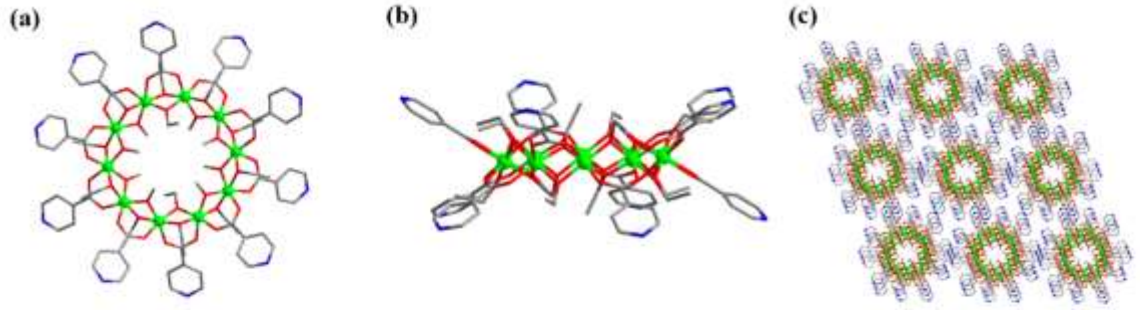

Supplementary Figure 63. The (a) top, (b) side, (c) packing view of **AlOC-79.** Hydrogen atoms are omitted for clarity. Color code: green Al, red O, blue N, grey C.

^{1}H NMR spectrum of the reaction mixture, where the green pentagram represents H on the product and the red asterisk represents H on CH_2Br_2.

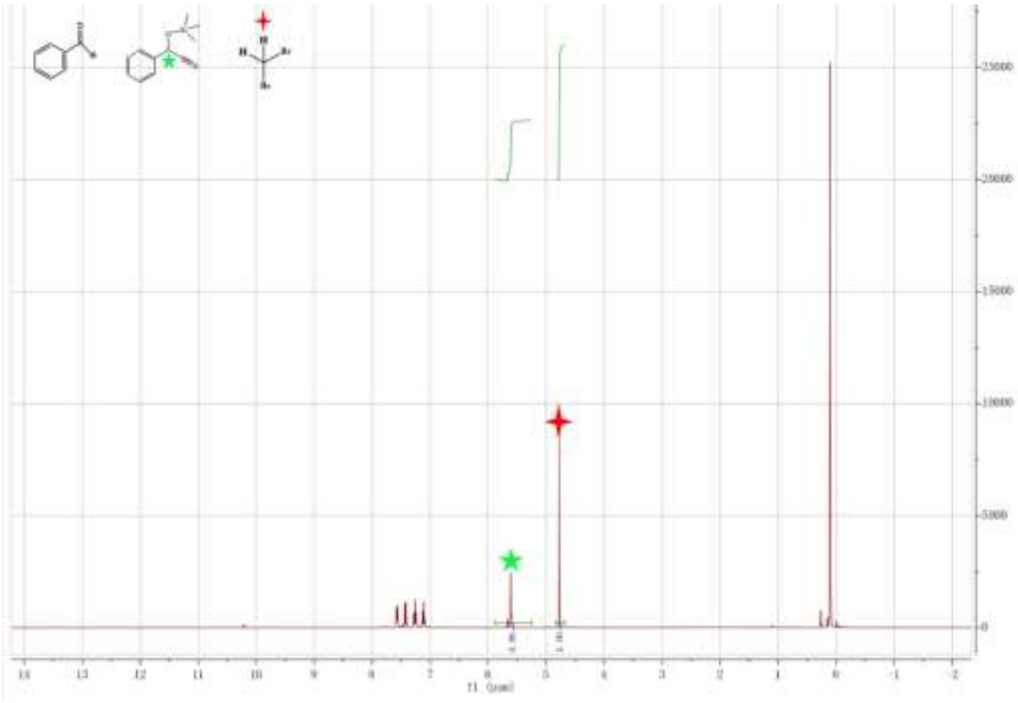

Supplementary Figure 64. ^{1}H NMR of the crude reaction mixture catalyzed by compound **AlOC-130** corresponding of Table 1, entry 5.

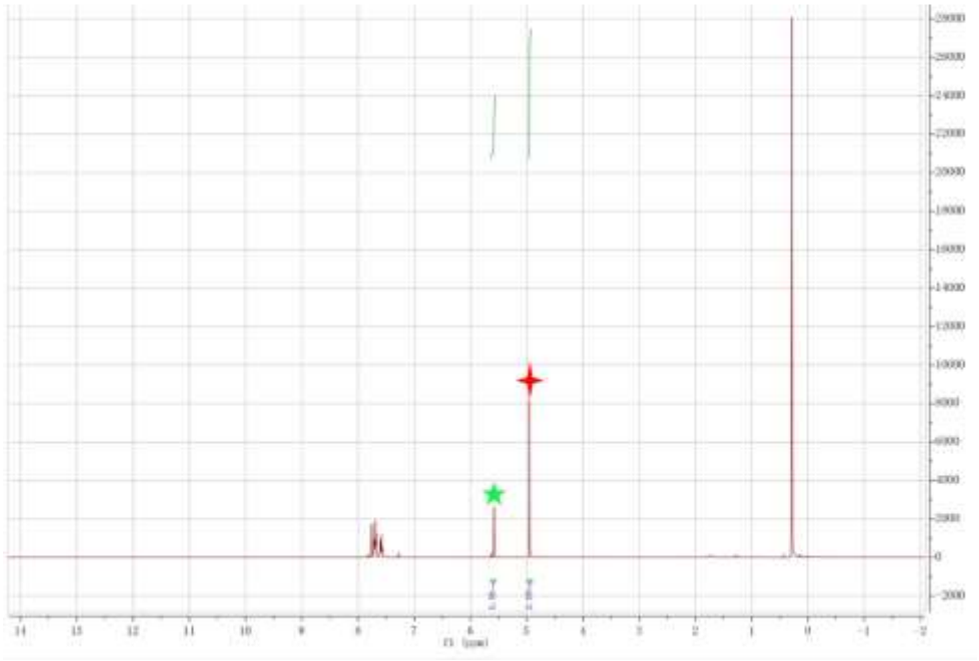

Supplementary Figure 65. ^{1}H NMR of the crude reaction mixture catalyzed by compound **AlOC-131** corresponding of Table 1, entry 6.

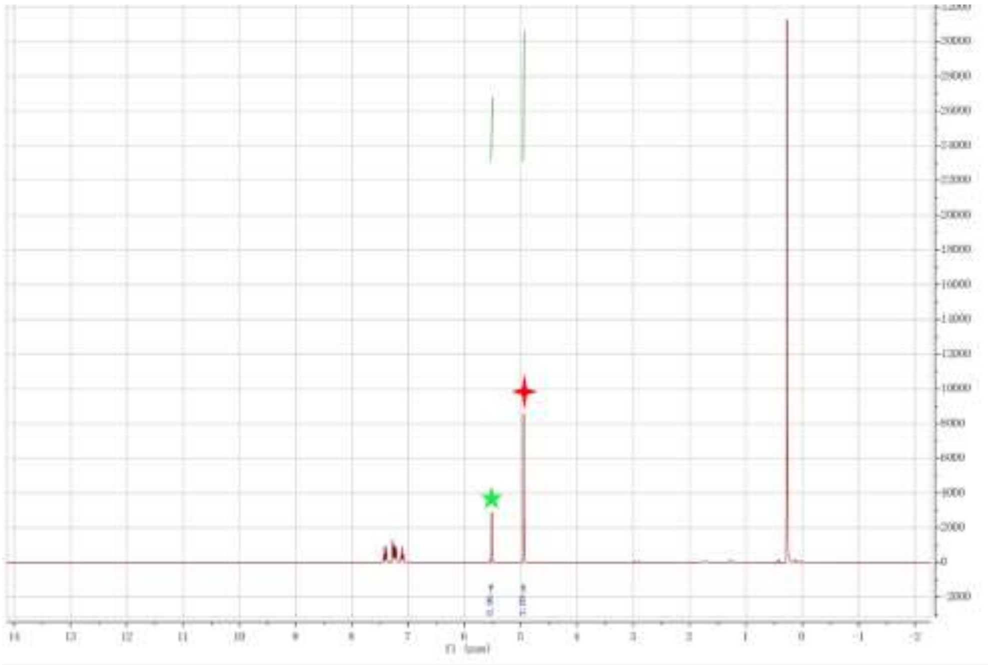

Supplementary Figure 66. ^{1}H NMR of the crude reaction mixture catalyzed by compound **AlOC-132** corresponding of Table 1, entry 7.

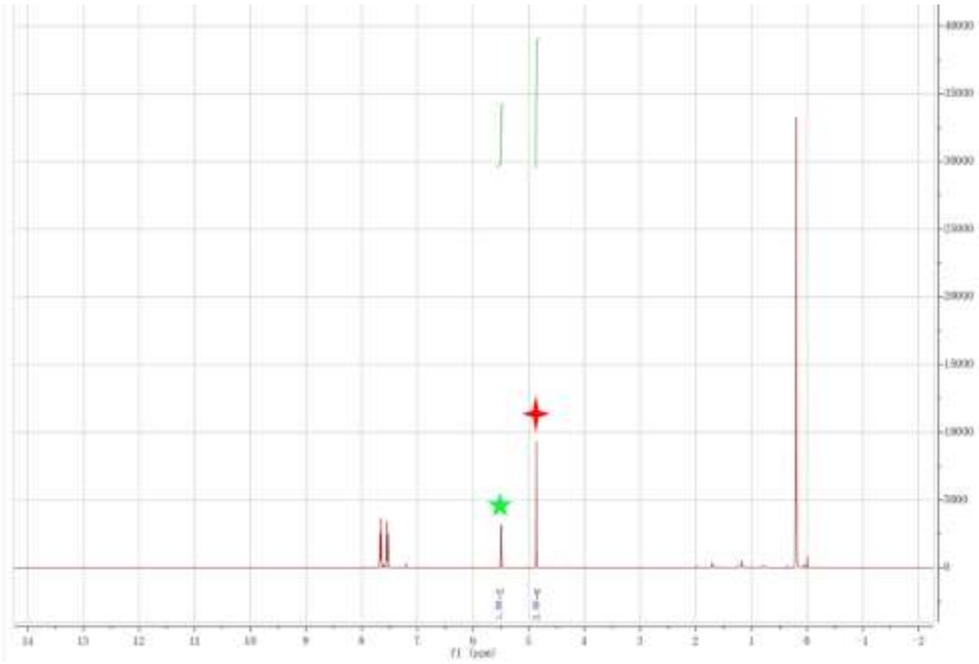

Supplementary Figure 67. ^{1}H NMR of the crude reaction mixture catalyzed by compound **AlOC-133** corresponding of Table 1, entry 8.

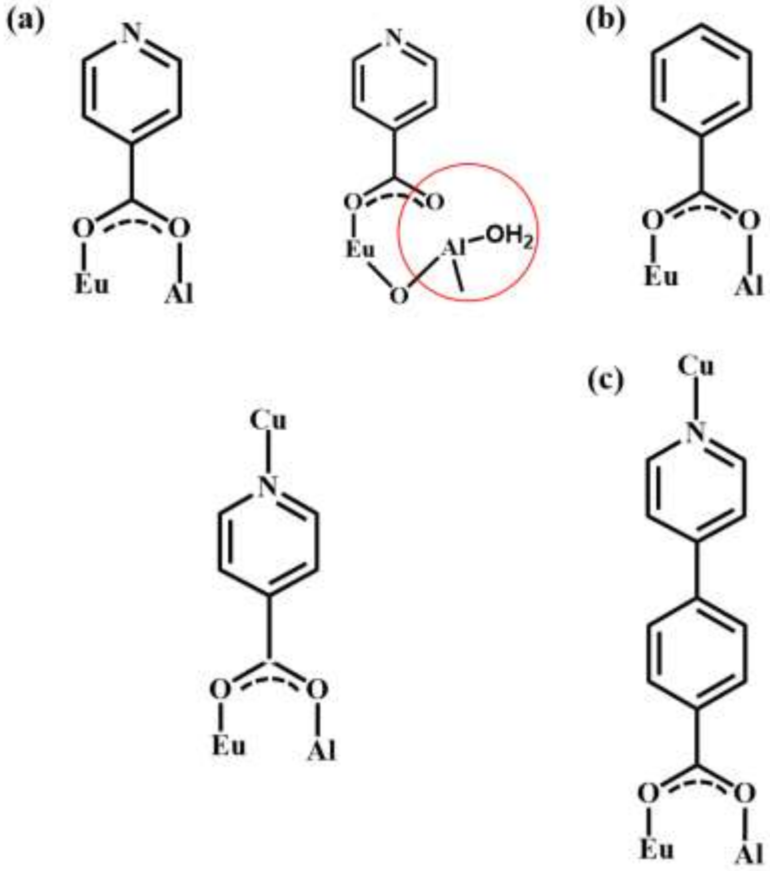

Supplementary Figure 68. The coordination mode of the organic ligand in the structure. (a) Saturation coordination of isonicotinic acid in compound **AlOC-131** (left). Coordination mode of isonicotinic acid with deletion, and the deletion is marked with a red circle (right). Coordination mode of isonicotinic acid in compound **AlOC-132** (bottom). (b) Mode of coordination of benzoic acid in compound **AlOC-130**. **(c)** Mode of coordination of elongated version of isonicotinic acid in compounds **AlOC-133**.

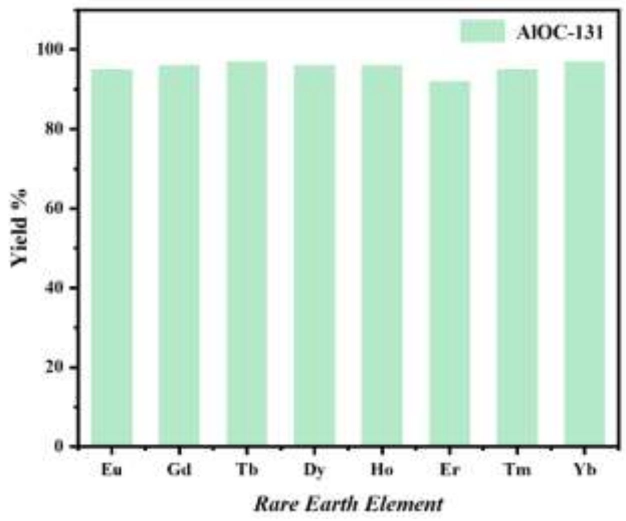

Supplementary Figure 69. Comparison of the catalytic effect of isostructures obtained by substitution of different rare earth metals by the compound **AlOC-131** in the cyanogenation of benzaldehyde with TMSCN.

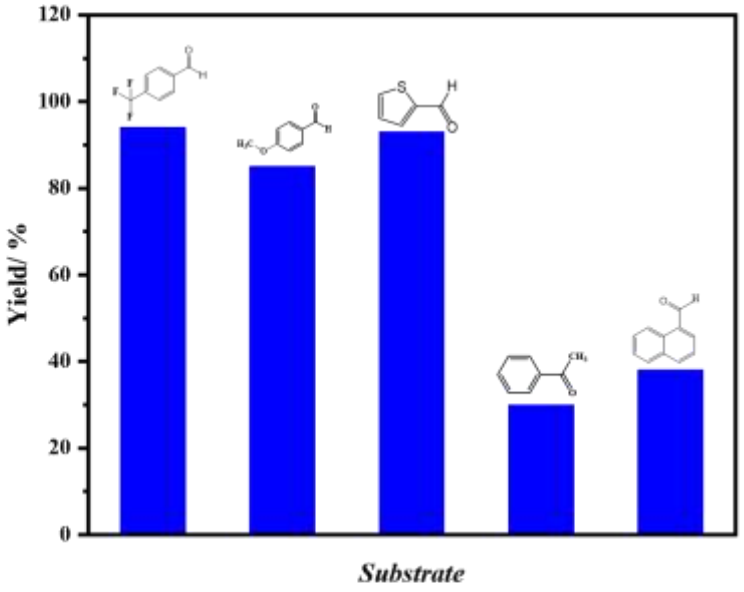

Supplementary Figure 70. The cyanosilylation of various carbonyl compounds with TMSCN.

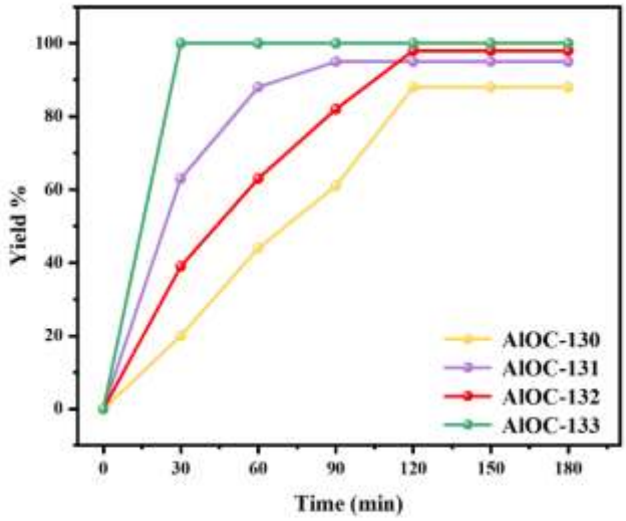

Supplementary Figure 71. Kinetic analysis of the benzaldehyde cyanosilylation reaction catalyzed by different heterometallic molecular rings (**AlOC-130** to **AlOC-133**).

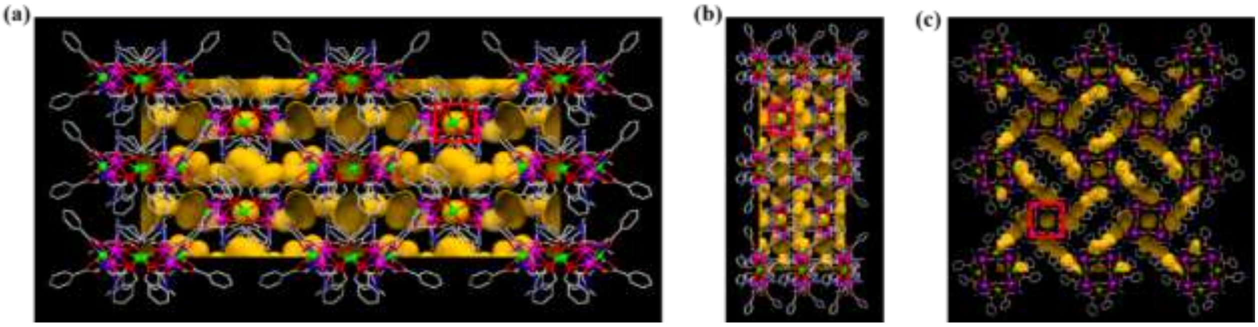

Supplementary Figure 72. Perspective view of the void space in **AlOC-130** along crystallographic axis. (a) a-axis; (b) b-axis; (c) c-axis. The central cavity of the molecular ring has been highlighted by the red box line.

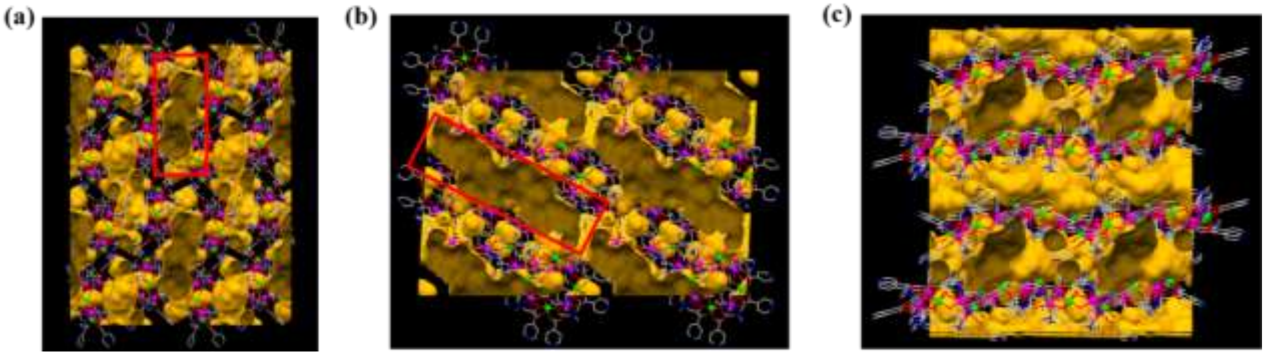

Supplementary Figure 73. Perspective view of the void space in **AlOC-131** along crystallographic axis. (a) a-axis; (b) b-axis; (c) c-axis. The one-dimensional channels formed by the stacking between molecular rings through hydrogen bonding have been highlighted by the red box lines.

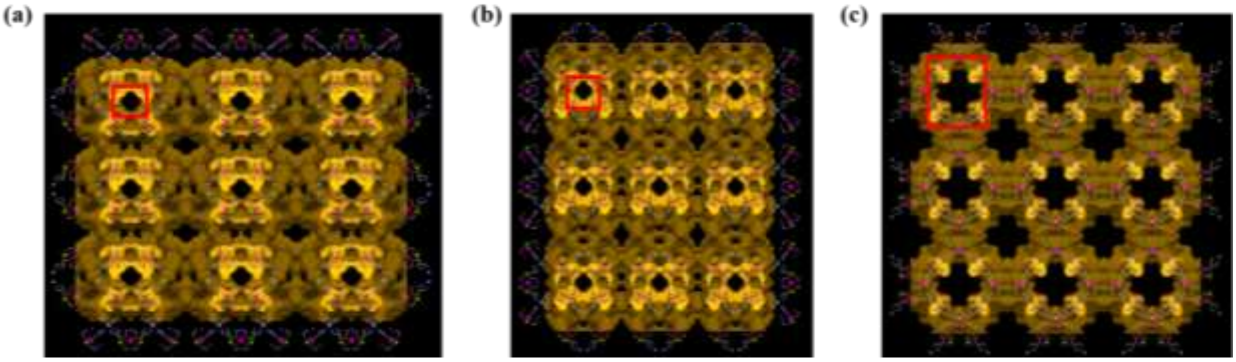

Supplementary Figure 74. Perspective view of the void space in **AlOC-132** along crystallographic axis. (a) a-axis; (b) b-axis; (c) c-axis. The 3D pores have been highlighted by the red box lines.

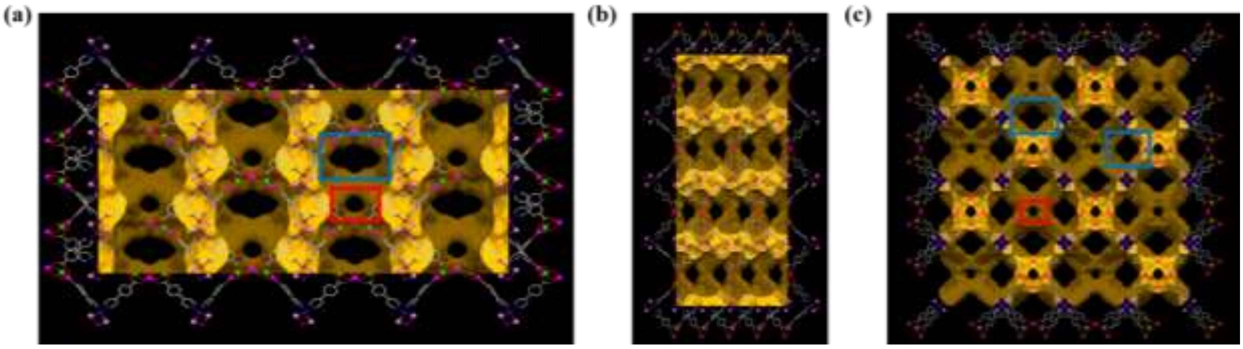

Supplementary Figure 75. Perspective view of the void space in **AlOC-133** along crystallographic axis. (a) a-axis; (b) b-axis; (c) c-axis. The ring center cavity has been marked with a red box line. One-dimensional channels have been marked by blue box lines.

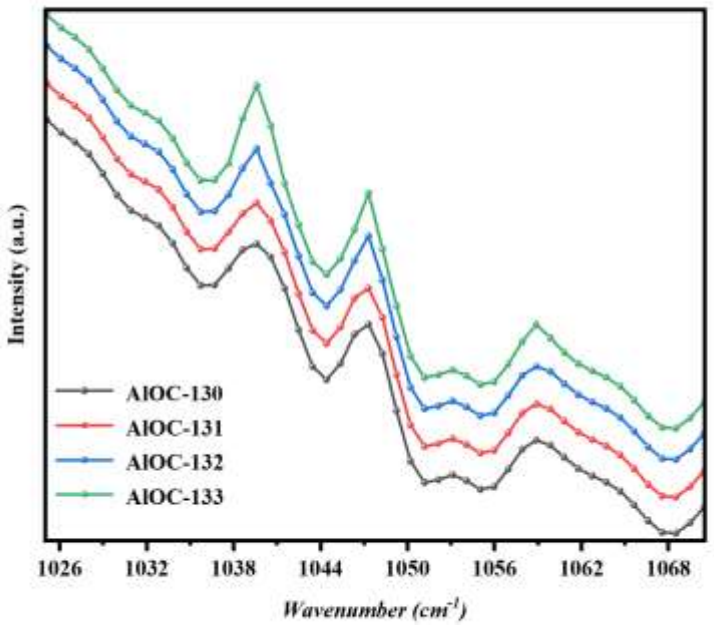

Supplementary Figure 76. FT-IR spectra of pyridine chemisorption on heterometallic molecular ring and its framework compounds.

4. Supplementary Tables

4.1 The solubility of heterometal rings in different solvents

Supplementary Table 1. The solubility of **AlOC-130** in solvents with different polarity.

solvent	polarity	solubleness [a]
petroleum ether	0.01	insoluble
n-hexane	0.06	insoluble
cyclohexane	0.1	insoluble
methylbenzene	2.4	soluble (solubility: <1mg/mL)
dichloromethane	3.4	insoluble
n-butylalcohol	3.9	insoluble
n-propanol	4.0	insoluble
tetrahydrofuran	4.2	insoluble
ethanol	4.3	insoluble
ethyl acetate	4.3	insoluble
isopropanol	4.3	insoluble
dioxane	4.8	insoluble
acetone	5.4	insoluble
acetonitrile	6.2	insoluble
dimethylformamide	6.4	insoluble
methanol	6.6	soluble (solubility: >1mg/mL)
dimethylsulfoxide	7.2	soluble (solubility: <1mg/mL)

Supplementary Table 2. The solubility of **AlOC-131** in solvents with different polarity.

solvent	polarity	solubleness [a]
petroleum ether	0.01	insoluble
n-hexane	0.06	insoluble
cyclohexane	0.1	insoluble
methylbenzene	2.4	soluble (solubility: ≈1mg/mL)
dichloromethane	3.4	soluble (solubility: ≈1mg/mL)
n-butylalcohol	3.9	insoluble
n-propanol	4.0	insoluble
tetrahydrofuran	4.2	insoluble
ethanol	4.3	insoluble
ethyl acetate	4.3	soluble (solubility: <1mg/mL)
isopropanol	4.3	insoluble
dioxane	4.8	soluble (solubility: <1mg/mL)
acetone	5.4	insoluble
acetonitrile	6.2	insoluble
dimethylformamide	6.4	insoluble
methanol	6.6	insoluble
dimethylsulfoxide	7.2	insoluble

[a] The solubility was evaluated by soaking 1 mg of crystals into 2 mL of solvent for 5 minutes.

4.2 Bond valence sum (BVS) calculations of heterometal rings.

Supplementary Table 3 BVS analysis for **AlOC-130**.

BVS Value	Bond distance	
Al1 3.1492	Al1-O4	1.943(6)
	Al1-O2	1.840(6)
	Al1-O5	1.964(6)
	Al1-O8[1]	1.871(6)
	Al1-O1[2]	1.866(6)
	Al1-O7	1.869(6)
Eu1 3.0800	Eu1-O6[1]	2.448(5)
	Eu1-O3	2.420(5)
	Eu1-O2	2.303(5)
	Eu1-O8[1]	2.393(5)
	Eu1-O1	2.315(5)
	Eu1-O7[1]	2.364(5)
	Eu1-N1	2.651(8)
	Eu1-N2[1]	2.641(6)

System code: [1]+Y, 1-X, 1-Z; [2]1-Y, +X, 1-Z.

Supplementary Table 4 BVS analysis for **AlOC-131**.

BVS Value	Bond distance		BVS Value	Bond distance	
Al1 3.1366	Al1-O9	1.861(5)	Al2 3.0865	Al2-O14	1.878(4)
	Al1-O5	1.865(5)		Al2-O15	1.999(5)
	Al1-O8	1.972(5)		Al2-O18	1.843(4)
	Al1-O6	1.844(5)		Al2-O19	1.947(5)
	Al1-O10	1.857(5)		Al2-O17	1.857(5)
	Al1-O11	1.970(5)		Al2-O13	1.881 (5)
Al3 3.1252	Al3-O21	1.878(5)	Al4 3.1351	Al4-O2	1.860(4)
	Al3-O27	1.999(4)		Al4-O31	1.869(5)
	Al3-O22	1.843(4)		Al4-O34	1.959(5)
	Al3-O25	1.947(5)		Al4-O30	1.851(5)
	Al3-O28	1.857(5)		Al4-O33	1.944(5)
	Al3-O26	1.881(5)		Al4-O1	1.878(5)
Eu1 3.0689	Eu1-O2	2.330(4)	Eu2 3.1111	Eu2-O14	2.336(4)
	Eu1-O4	2.450(4)		Eu2-O9	2.337(4)
	Eu1-O5	2.327(4)		Eu2-O10	2.370(4)
	Eu1-O6	2.378(4)		Eu2-O16	2.402(4)
	Eu1-O7	2.431(5)		Eu2-O13	2.349(4)
	Eu1-O1	2.338(4)		Eu2-O12	2.431(4)
	Eu1-N2	2.636(5)		Eu2-N5	2.621(6)
	Eu1-N1	2.644(6)		Eu2-N6	2.625(6)
Eu3 2.9898	Eu3-O21	2.355(4)	Eu4 2.9596	Eu4-O31	2.385(4)
	Eu3-O20	2.418(4)		Eu4-O27	2.405(4)
	Eu3-O18	2.424(4)		Eu4-O30	2.354(4)
	Eu3-O22	2.389(4)		Eu4-O32	2.509(4)
	Eu3-O17	2.366(4)		Eu4-O26	2.347(4)
	Eu3-O24	2.341(4)		Eu4-O29	2.457(5)
	Eu3-N9	2.655(5)		Eu4-N13	2.682(5)
	Eu3-N10	2.662(5)		Eu4-N14	2.703(6)

Supplementary Table 5 BVS analysis for **AlOC-132**.

BVS Value	Bond distance	
Al05 2.9629	Al05-O07^{4}	1.84(3)
	Al05-O07	1.84(3)
	Al05-O08^{4}	1.93(3)
	Al05-O08	1.93(3)
	Al05-O09	1.98(3)
	Al05-O09^{4}	1.98(3)
Eu01 2.7390	Eu01-O6^{1}	2.46(3)
	Eu01-O06	2.46(3)
	Eu01-O07	2.30(3)
	Eu01-O07^{1}	2.30(3)

	Eu01-O08	2.35(3)
	Eu01-O08^{1}	2.35(3)
	Eu01-N0A	2.70(4)
	Eu01-N00E	2.43(8)
Cu04 1.2133	Cu04-N00B	1.97(4)
	Cu04-N00B^{3}	1.97(3)
	Cu04-I02	2.60(11)
	Cu04-I03	2.67(11)

System code:^{1}+X, 2-Y, +Z; ^{2}+X, +Y, 1-Z; ^{3}+Y, +X, +Z; ^{4}3/2-Y, 3/2-X, 1/2-Z.

Supplementary Table 6 BVS analysis for **AlOC-133**.

BVS Value	Bond distance		BVS Value	Bond distance	
Al 2.9073	Al1-O2	1.96(2)	Cu 1.0975	Cu1-I1	2.675(5)
	Al1-O3	1.90(2)		Cu1^{3}-I1	2.628(5)
	Al1-O4	1.87(2)		Cu1-N	2.05(3)
	Al1-O5	1.95(2)		Cu1-N24^{4}	1.99(3)
	Al1-O7	1.87(2)			
	Al1-O8	1.81(2)			
Eu1 2.5000	Eu1-O6^{1}	2.46(2)	Eu2 2.6513	Eu2-O1	2.45(2)
	Eu1-O6	2.46(2)		Eu2-O1^{2}	2.45(2)
	Eu1-O7	2.343(18)		Eu2-O3	2.37(2)
	Eu1-O7^{1}	2.343(18)		Eu2-O3^{2}	2.37(2)
	Eu1-O8^{1}	2.40(2)		Eu2-O4^{2}	2.32(16)
	Eu1-O8	2.40(2)		Eu2-O4	2.32(17)
	Eu1-N2	2.54(5)		Eu2-N1	2.56(4)
	Eu1-N4	2.63(4)		Eu2-N3	2.70(4)

System code: ^{1}+X, +Y, 1/2-Z; ^{2}1-X, +Y, +Z; ^{3}3/2-X, 3/2-Y, 1-Z; ^{4}3/2-X, 1/2-Y, 1-Z.

4.3 Hydrogen bond parameters

Supplementary Table 7 Hydrogen bond parameters for **AlOC-130**.

D-H···A	d(D-H) (Å)	d(H-A) (Å)	d(D-A) (Å)	<(DHA)
C1-H1B···O8	0.97	2.40	2.988	119
C5-H5A···O7	0.97	2.38	2.984	120
C20-H20B···O4	0.97	2.33	2.875	115

Supplementary Table 8 Hydrogen bond parameters for **AlOC-131**.

D-H···A	d(D-H) (Å)	d(H-A) (Å)	d(D-A) (Å)	<(DHA)
O25-H25A···O23	0.87	1.85	2.656	154
O25-H25B···N3^{i}	0.87	1.91	2.715	154
O35-H35A···O1	0.87	2.50	2.862	106
O35-H35A···O30	0.87	2.50	2.808	102
O35-H35A···O36^{ii}	0.87	1.99	2.683	136
O35-H35B···O21	0.87	2.33	3.160	159

O35-H35B···O26	0.87	2.32	2.768	112
O36-H36A···O5^{ii}	0.87	2.29	3.151	172
O36-H36A···O9	0.87	2.57	3.009	112
O36-H36B···O14	0.87	2.07	2.934	169
C5-H5B···O33	0.99	2.37	2.951	117
C6-H6B···O10	0.99	2.39	3.010	120
C28-H28B···O6	0.99	2.38	3.001	120
C32-H32A···O18	0.99	2.43	3.031	119
C42-H42···N12^{i}	0.99	2.60	3.230	124
C49-H49A···O35	0.99	2.53	3.243	129
C53-H53C···O20	0.99	2.60	3.183	119
C55-H55A···O28	0.99	2.48	2.945	109
C68-H68A···O25	0.99	2.37	2.993	121
C70-H70C···O32	0.99	2.51	3.140	122
C75-H75B···O29	0.99	2.57	3.203	122
C77-H77A···O2	0.99	2.26	2.839	116
C91-H91B···O32	0.99	2.55	3.106	116
C98-H98A···O35	0.99	2.57	3.170	119
C98-H98B···O34	0.99	2.33	2.930	118

Symmetric code: (i) -1+x, y, z; (ii) 1/2+x, 1/2-y, 1/2+z.

4.4 Summary of catalysts used in the catalytic reaction of cyanogenation of aldehydes.

Supplementary Table 9. Summary of catalysts used in the catalytic reaction of cyanogenation of aldehydes.

Catalyst	Reaction conditions	Reaction time and yield	Major active contribution	TON	Ref.
1	Benzaldehyde (1.5 mmol) TMSCN (3 mmol) CH_2Cl_2 (5 mL) 303K	9h 98%	Porous Mn^{II} site	9	[8]
1	Benzaldehyde (0.5 mmol) TMSCN (1.5 mmol) Solvent-free 323K	4h 99.2%	Rare earth metal ions	49.6	[9]
2		4h 97.1%		48.5	
3		4h 97.8%		48.9	
4		4h 66.7%		33.4	
5		4h 70.4%		35.2	
6		4h 68.1%		34.1	
1(Nd)	Benzaldehyde (0.5 mmol) TMSCN (1 mmol) CH_2Cl_2 (5 mL) 303K	2h 99%	Rare earth metal ions	22	[10]
6 (Ho)		2h 76%		17	
7 (Er)		5h 61%		14	
8 (Yb)		5h 57%		13	
1	Benzaldehyde (2 mmol) TMSCN (3 mmol) Solvent-free 303K	1h 99%	Aluminum Hydride	198	[11]
		5min 99%		999	
3	Benzaldehyde (1 mmol) TMSCN (1.2 mmol) Solvent-free	10 min 99%	Cationic Aluminium Complex	99	[12]
		25 min 99%		198	

2	303K	60 min 99%		99	
$[LAlMe]^+[MeB(C_6F_5)_3]^-$	Benzaldehyde (1 mmol) TMSCN (1 mmol) Solvent-free 303K	5 min 98%	Organoaluminum Cation	1960	[13]
Y-POM	Benzaldehyde (0.5 mmol) TMSCN (0.75 mmol) Solvent-free 303K	6h 75%	Rare earth metal ions	75	[14]
Eu-POM		6h 76%		76	
Tb-POM		6h 70%		70	
Yb-POM		6h 71%		71	
Sm-POM		6h 47%		47	
La-POM		6h 49%		49	
Pr-POM		6h 46%		46	
**AlOC-130**	Benzaldehyde (0.5 mmol) TMSCN (1 mmol) CH_2Cl_2 (5 mL) 303K	2h 88.0%	Synergistic catalysis by Al^{III} and Ln^{III}	58.7	This work
**AlOC-131**		2h 95.5%		63.3	
**AlOC-132**		2h 98.2%	Synergistic catalysis by Al^{III}, Ln^{III} and Cu^{I}	64	
**AlOC-133**		0.5h 99.9%		66.7	
MIL-47 (V)	Benzaldehyde (1 mmol) TMSCN (1.2 mmol) Solvent-free 303K	3h 46%	V^{IV} site	46	[15]
MIL-53 (Al)		3h 26%	Al^{III} site	26	
MIL-101 (Cr)		3h 96%	Cr site	96	
Uio-66 (Zr)		3h 68%	Zr site	68	
Cd-MDIP	Benzaldehyde (0.5 mmol) TMSCN (1.2 mmol) CH_2Cl_2 (2 mL) 303K	2h 10%	Cd site	20	[16]
Cd-MDIP⊃Fe^{3+}		2h 97%	Cd and Fe^{3+} site	194	
AlOC-58NC	Benzaldehyde (0.5 mmol) TMSCN (1 mmol) CH_2Cl_2 (5 mL) 303K	2h 80%	Al^{III} site	53.3	[7]
AlOC-59NT		2h 92%	Al^{III} site	61.3	

4.5 Crystallographic data of heterometal rings.

Supplementary Table S10. Experimental single-crystal X-ray data for **AlOC-130** and **AlOC-131.**

	**AlOC-130**	**AlOC-131**
Empirical formula	$Al_4Eu_4C_{96}N_8O_{32}H_{128}$	$Al_4Eu_4C_{88}N_{16}O_{35}H_{126}$
Formula weight	2621.85	2683.81
Temperature / K	100.00(10)	100.00(10)
Crystal system	tetragonal	monoclinic
Space group	P-4$2_1$c	$P2_1/n$
a [Å]	23.3687(10)	18.9563(2)
b [Å]	23.3687(10)	21.8313(2)
c [Å]	9.8818(10)	27.7034(4)
α [◦]	90	90
β [◦]	90	93.1440(10)
γ [◦]	90	90
V [Å ^{3}]	5396.41(7)	11447.5(2)

Z	2	4
ρcalcd [g cm^{-3}]	1.599	1.553
μ [mm^{-1}]	12.916	12.213
F (000)	2592.0	5373.0
	-30≤ h ≤30	-24 ≤ h ≤ 24
Index ranges	-30 ≤ k ≤ 29	-28 ≤ k ≤ 28
	-12 ≤ l ≤12	-34 ≤ l ≤ 36
Reflections collected	44962	93223
Independent refs [R_{int}]	5993[0.0674]	25769 [0.0458]
date/restraints/parameters	5993/49/337	25769/61/1447
Goodness-of-fit on F^2	1.051	1.110
R_1[a], wR_2[b] [I>2σ(I)]	0.0386, 0.0985	0.0592, 0.1145
R_1, wR_2 [all data]	0.0422, 0.1009	0.0719, 0.1184
CCDC number	2288338	2288339

[a]$R_1 = \Sigma||F_o|-|F_c||/\Sigma|F_o|$. [b]$wR_2 = \{\Sigma[w(F_o^2-F_c^2)^2]/\Sigma[w(F_o^2)^2]\}^{1/2}$

Supplementary Table S11. Experimental single-crystal X-ray data for **AlOC-132** and **AlOC-133.**

	**AlOC-132**	**AlOC-133**
Empirical formula	$Al_4Eu_4Cu_4I_4C_{88}N_{16}O_{32}H_{120}$	$Al_4Eu_4Cu_4I_4C_{136}N_{16}O_{32}H_{152}$
Formula weight	3391.56	4000.33
Temperature / K	100.00(19)	100.00(2)
Crystal system	tetragonal	orthorhombic
Space group	*I*4/mmm	*C*mcm
a [Å]	31.674 (4)	41.3594(17)
b [Å]	31.674 (4)	17.9180(8)
c [Å]	25.087 (5)	40.8506(15)
α [◦]	90	90
β [◦]	90	90
γ [◦]	90	90
V [Å^3]	25169(7)	30273(2)
Z	4	4
ρcalcd [g cm^{-3}]	0.867	0.851
μ [mm^{-1}]	11.675	9.746
F (000)	6144.0	7584.0
	-26 ≤ h ≤ 32	-38 ≤ h ≤ 51
Index ranges	-27 ≤ k ≤ 17	-22 ≤ k ≤ 19
	-20 ≤ l ≤ 13	-51 ≤ l ≤ 49
Reflections collected	11286	50486
Independent refs [R_{int}]	3144 [0.1123]	14950[0.1753]
date/restraints/parameters	3144/68/161	14950/103/454
Goodness-of-fit on F^2	0.926	1.272

R_1[a], wR_2[b] [$I>2\sigma(I)$]	0.0998, 0.2659	0.1618, 0.4031
R_1, wR_2 [all data]	0.1931, 0.3463	0.2259, 0.4465
CCDC number	2288340	2288341

[a] $R_1 = \Sigma||F_o|-|F_c||/\Sigma|F_o|$. [b] $wR_2 = \{\Sigma[w(F_o^2-F_c^2)^2]/\Sigma[w(F_o^2)^2]\}^{1/2}$

5. Supplementary Reference.

[1] G. M. Sheldrick, *Acta Cryst. C* **2015**, *71*, 3-8.

[2] Y.-S. Jin, X. Wang, N. Zhang, C.-M. Liu, H.-Z. Kou, *Cryst. Growth Des.* **2022**, *22*, 1263-1269.

[3] S. Chen, V. Mereacre, C. E. Anson, A. K. Powell, *Dalton Trans.* **2016**, *45*, 9336-9344.

[4] S. K. Langley, C. M. Forsyth, B. Moubaraki, K. S. Murray, *Dalton Trans.* **2015**, *44*, 912-915.

[5] L. Geng, C. H. Liu, S. T. Wang, W. H. Fang, J. Zhang, *Angew. Chem. Int. Ed.* **2020**, *59*, 16735-16740.

[6] S. S. Yao, W. H. Fang, Y. Sun, S. T. Wang, J. Zhang, *J. Am. Chem. Soc.* **2021**, *143*, 2325-2330.

[7] D. Luo, H. Xiao, M. Y. Zhang, S. D. Li, L. He, H. Lv, C. S. Li, Q. P. Lin, W. H. Fang, J. Zhang, *Chem. Sci.* **2023**, *14*, 5396-5404.

[8] S. Horike, M. Dincaˇ, K. Tamaki, J. R. Long, *J. Am. Chem. Soc.* **2008**, *130*, 5854–5855.

[9] H. An, Y. Zhang, Y. Hou, T. Hu, W. Yang, S. Chang, J. Zhang, *Dalton Trans.* **2018**, *47*, 9079-9089.

[10] M. Gustafsson, A. Bartoszewicz, B. Martín-Matute, J. Sun, J. Grins, T. Zhao, Z. Li, G. Zhu, X. Zou, *Chem. Mater.* **2010**, *22*, 3316-3322.

[11] Z. Yang, M. Zhong, X. Ma, S. De, C. Anusha, P. Parameswaran, H. W. Roesky, *Angew. Chem. Int. Ed.* **2015**, *54*, 10225-10229.

[12] M. K. Sharma, S. Sinhababu, G. Mukherjee, G. Rajaraman, S. Nagendran, *Dalton Trans.* **2017**, *46*, 7672-7676.

[13] S. Rawat, M. Bhandari, B. Prashanth, S. Singh, *ChemCatChem* **2020**, *12*, 2407-2411.

[14] S. Li, Y. Zhou, Q. Peng, R. Wang, X. Feng, S. Liu, X. Ma, N. Ma, J. Zhang, Y. Chang, Z. Zheng, X. Chen, *Inorg. Chem.* **2018**, *57*, 6624-6631.

[15] Z. Zhang, J. Chen, Z. Bao, G. Chang, H. Xing, Q. Ren, *RSC Advances* **2015**, *5*, 79355-79360.

[16] Y. Li, Z. Chang, F. Huang, P. Wu, H. Chu, J. Wang, *Dalton Trans.* **2018**, *47*, 9267-9273.
